# Chromosome 1 licenses chromosome 2 replication in *Vibrio cholerae* by doubling the *crtS* gene dosage

**DOI:** 10.1371/journal.pgen.1007426

**Published:** 2018-05-24

**Authors:** Revathy Ramachandran, Peter N. Ciaccia, Tara A. Filsuf, Jyoti K. Jha, Dhruba K. Chattoraj

**Affiliations:** Laboratory of Biochemistry and Molecular Biology, Center for Cancer Research, National Cancer Institute, National Institutes of Health, Bethesda, Maryland, United States of America; University of Toronto, UNITED STATES

## Abstract

Initiation of chromosome replication in bacteria is precisely timed in the cell cycle. Bacteria that harbor multiple chromosomes face the additional challenge of orchestrating replication initiation of different chromosomes. In *Vibrio cholerae*, the smaller of its two chromosomes, Chr2, initiates replication after Chr1 such that both chromosomes terminate replication synchronously. The delay is due to the dependence of Chr2 initiation on the replication of a site, *crtS*, on Chr1. The mechanism by which replication of *crtS* allows Chr2 replication remains unclear. Here, we show that blocking Chr1 replication indeed blocks Chr2 replication, but providing an extra *crtS* copy in replication-blocked Chr1 permitted Chr2 replication. This demonstrates that unreplicated *crtS* copies have significant activity, and suggests that a role of replication is to double the copy number of the site that sufficiently increases its activity for licensing Chr2 replication. We further show that *crtS* activity promotes the Chr2-specific initiator function and that this activity is required in every cell cycle, as would be expected of a cell-cycle regulator. This study reveals how increase of gene dosage through replication can be utilized in a critical regulatory switch.

## Author summary

The timing of DNA replication initiation is controlled in all growing cells. The conventional wisdom is that in bacteria such as *E*. *coli*, initiation occurs when sufficient active form of the initiator protein accumulates. The same scenario possibly applies to *V*. *cholerae* Chr1, whose replication in turn determines the timing of replication of its second chromosome, Chr2. This is because Chr2 replication initiation depends on the replication of a site, *crtS*, in Chr1. Here we show that the *crtS* site has significant activity towards triggering Chr2 replication without itself being replicated. We propose that the doubling of *crtS* copy number, normally afforded by replication, allows accumulation of enough active Chr2-specific initiator to trigger initiation. The study provides a novel example of how the timing of a gene function can be controlled by the timing of its duplication.

## Introduction

Nearly 10% of bacteria from diverse phyla distribute their genetic information into more than one chromosome [1]. The presence of multipartite genomes in bacteria provides a challenge to understand the mechanisms that determine timely replication and segregation of all chromosomes of a genome prior to cell division. Failures in these processes can cause genome instability, slowed cell growth and cell death [2].

Chromosome dynamics in bacteria with multipartite genomes is well studied in the human pathogen *Vibrio cholerae*. All members of the *Vibrio* genus possess two circular, unequal-sized chromosomes, Chr1 and Chr2. The size of Chr1 is fairly conserved (3.0 to 3.4 Mb) whereas there is greater diversity in the size of Chr2 (0.8 to 2.4 Mb) [3]. The replication of the two chromosomes is regulated differently [4]. The regulation of Chr1 is similar to that of the well-studied *Escherichia coli* chromosome and is mediated through the universal initiator of bacterial replication, DnaA [5]. Chr2 replication is regulated by its specific initiator RctB, which is highly conserved in the *Vibrionaceae* family [4, 6].

The regulation of Chr2 replication is reminiscent of the copy number control of some plasmids [7, 8]. The salient feature of Chr2 replication origin is the presence of an array of tandem repeats of 12-mers where the initiator RctB binds. This feature of the origin is common in members of the iteron family of plasmids [5]. Additionally, the central region of RctB is structurally homologous to plasmid initiators [9, 10]. The Chr2 replicon, however, has many additional features that distinguish it from its presumed plasmid progenitor. For example, in addition to 12-mers, RctB binds to a second kind of site, 39-mers, which inhibits initiation [11]. Another mechanism to inhibit initiation is built-in to the 12-mers in the form of adenine methylation sequences (GATC), which become hemi-methylated following replication initiation and allow binding of the SeqA protein [5, 12]. Inhibition of replication initiation upon SeqA binding also occurs in the origins of the *E*. *coli* chromosome and Chr1. Thus, although seemingly plasmid-derived, the complexity of Chr2 replication control is comparable to those of other well-studied bacterial chromosomes, which is apparently required to maintain once-per-cell-cycle replication [8].

Replication of Chr2 initiates after Chr1 [13]. The lag in initiation time is such that both chromosomes terminate replication at approximately the same time. The basis of the termination synchrony is due to a novel replication timing mechanism. A unique RctB-binding site, *crtS* (Chr2 replication triggering site), was discovered in Chr1 within a 153-bp locus [14]. Chr2 replication initiation occurs after the Chr1 replication fork moves past the *crtS* site [15]. This appears to be a conserved feature in most *Vibrio* species [16]. The genomic location of *crtS* is such that it appropriately defers Chr2 replication for termination synchrony. Moving *crtS* to ectopic sites changes the timing of Chr2 replication in accordance with the time at which the site is duplicated, which indicates that *crtS* locus needs to be replicated for Chr2 replication initiation [15]. Thus far, this is the only genetic scheme known for coordinating replication timing of individual chromosomes in bacteria with multipartite genomes.

Supplying *crtS* on a multicopy plasmid increases replication of mini-Chr2 (plasmids with Chr2 replication origin, *ori2*) in *E*. *coli* when RctB is provided, although some mini-Chr2 replication is seen in the absence of *crtS* as well [14]. Increasing *crtS* copy number in Chr1 also increases Chr2 replication in *V*. *cholerae* [15]. These results indicate that *crtS* function is limiting for Chr2 replication [14, 15]. This is further supported by the fact that deletion of *crtS* leads to chromosomal fusion or selection of suppressor mutations [15]. The role of *crtS* thus appears crucial for Chr2 replication in *V*. *cholerae*.

The only clue as to how *crtS* might function comes from the *in vivo* finding that in its presence, RctB binding to 12-mers increases and to 39-mers decreases, both of which are favorable for initiation [14]. These results suggest that *crtS* might remodel RctB into a more proficient initiator, which is further supported by the ability of *crtS* to partially substitute the DnaK and DnaJ chaperones required for RctB function. Overall, these results indicate that *crtS* replication is required to trigger Chr2 replication and the mechanism of triggering is through remodeling of RctB. However, how replication helps *crtS* function remains an open question. Replication can serve by doubling the site dosage and the cumulative activity of two sites could suffice to remodel enough RctB to trigger Chr2 replication initiation. It is also possible that the passage of the replication fork across *crtS* changes the topology of the site to make it more active. Replication could cause *crtS* to be transiently hemi-methylated or single-stranded, or could clear out possible bound inhibitory factors such that the site can interact with RctB appropriately. To address some of these possible roles of replication in *crtS* function, here we devised an approach to conditionally block Chr1 replication in *V*. *cholerae* and investigated the consequences towards triggering Chr2 replication initiation.

We conclude that the *crtS* locus has significant activity without replication, and doubling the *crtS* gene dosage, which is normally accomplished by Chr1 replication, suffices to activate Chr2 replication. The product but not the process of replication thus appears obligatory for *crtS* function. The unreplicated *crtS* locus appears to promote the initiator function of RctB, as in replication-permissive conditions. Our finding that *crtS* has activity without replication is expected to simplify mechanistic studies on how *crtS* triggers Chr2 replication initiation.

## Results

### Blocking Chr1 replication blocks Chr2 replication

**Fig 1:**
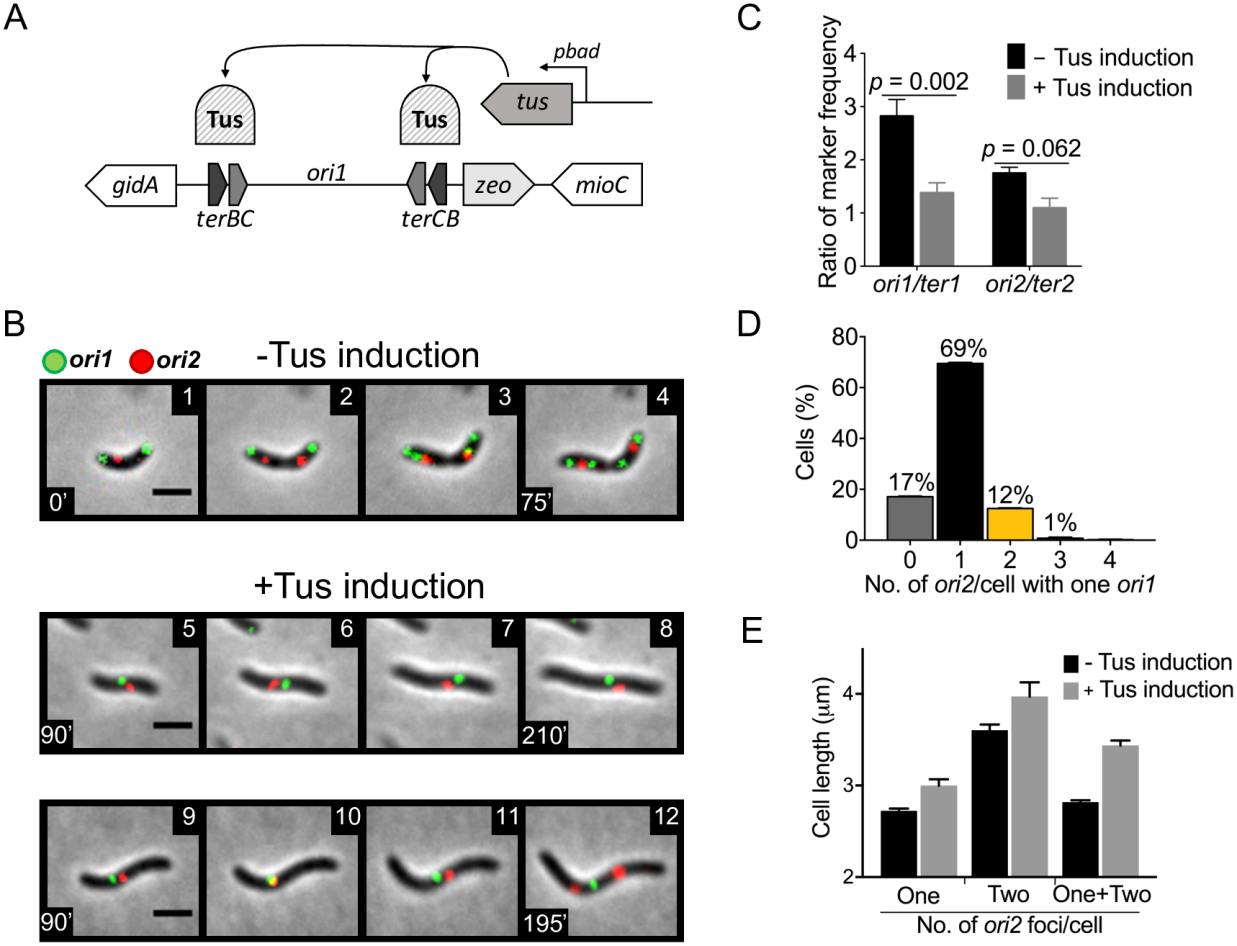
Blocking replication of Chr1 blocks Chr2 replication. (A) Scheme for blocking Chr1 replication by induction of *ter*-binding Tus protein from *pbad* promoter. (B) Time-lapse microscopy of *V*. *cholerae* (CVC3022) showing *ori1* (green) and *ori2* (red) foci when cells were grown either without inducer (panels 1–4) or with 0.2% inducer (arabinose) to induce Tus for blocking Chr1 replication (panels 5–12). Without inducer, cells were born with two *ori1* foci and one *ori2* focus (panel 1). Upon cell growth, the *ori2* focus duplicates (panel 2), followed by duplication of *ori1* before cell division (panels 3,4). With Tus induction, many cells show one *ori1* focus, indicating a block of Chr1 replication (panel 5). In most of these cells, the *ori2* focus remains single, indicating that Chr2 replication is blocked (panels 6–8). In a few cells, the *ori2* focus was seen to divide even as the *ori1* focus remained single, (panels 9–12). Scale bars, 2 μm. (C) Marker frequency analysis by qPCR. Markers analyzed were *ori1*, *ter1*, *ori2* and *ter2* of *V*. *cholerae* grown in LB in the absence (black) or presence (grey) of 0.2% arabinose for 150 min. The induction reduced the *ori1/ter1* and *ori2/ter2* values. Data represents mean ± standard error of mean (SEM) from three biological replicates, each performed in triplicate. Statistical significance was calculated using a Student’s *t*-test. (D) Histogram of *ori2* foci number per cell (x-axis) as percentages in cells that have one *ori1* focus (y-axis). The cells with no visible *ori2* focus (grey bars, ~17%) are possibly due to weak tdTomato fluorescence. Data represents mean ± SEM of percentages calculated from three biological replicates each with at least 100 cells (Σn = total cells counted = 522). (*E*) Mean length of cells with one *ori1* focus, two *ori2* foci and one or two *ori2* foci. Note that the cells with two *ori2* foci are longer, revealing that with accumulation of mass Chr2 can overcome its dependence on Chr1 replication. Error bars denote SEM.

In *V*. *cholerae*, blocking of Chr2 replication initiation does not prevent Chr1 replication or cell division, indicating that there is no checkpoint to ensure completion of genome duplication before cell division [17]. Here, we blocked Chr1 replication and tested its effect on the replication of Chr2. In the earlier study, Chr2 replication was blocked by adding an excess of 39-mer sites that specifically inhibited Chr2 replication initiation. Because there is no known Chr1-specific inhibitor of initiation, we utilized the Tus-*ter* replication fork barrier system of *E*. *coli* to conditionally block Chr1 replication [18]. Two *E*. *coli ter* sites were inserted on each flank of the Chr1 origin of replication, *ori1*, and the *ter*-binding Tus protein of *E*. *coli* was supplied under control of the arabinose-inducible promoter, *pbad* (Fig 1A). By observing origin proximal fluorescent markers, we could simultaneously visualize Chr1 and Chr2 replication by microscopy. The *ori1* region was marked by GFP- P1ParB fluorescent protein bound to a P1*parS* site inserted at +135 kb from the origin. The *ori2* region was marked by tdTomato-pMTParB fluorescent protein bound to a pMT*parS* site inserted at +40 kb from the origin [17]. The genes encoding the proteins were chromosomally inserted and expressed under control of the IPTG-inducible promoter, *ptac*.

Time-lapse microscopy showed that under our growth conditions, *V*. *cholerae* cells were born with two *ori1* foci, one near each cell pole, and a single *ori2* focus near the cell center (Fig 1B, panel 1). Subsequently, the *ori2* focus duplicated and occupied quarter-cell positions, while the two *ori1* foci duplicated prior to completion of cell division (panels 2–4). This scenario is indicative of multi-fork replication of Chr1 that results when the time to complete duplication of the chromosome exceeds the cell generation time [19, 20]. Following Tus induction to block Chr1 replication, cells contained only 1 *ori1* focus (panels 5–8). The focus did not duplicate as the cells lengthened for the duration of the time-lapse experiment (equivalent to at least two generations in liquid medium under replication-permissive conditions). Although the cells continued to lengthen, they failed to divide, indicative of blockage of Chr1 replication [21]. The localization of *ori1* was also not polar, unlike under normal growth (S1 Fig). The block in Chr1 replication was confirmed by qPCR where the *ori1/ter1* ratio was seen to reduce from 2.8 to 1.4 at 150 min after Tus induction (Fig 1C). In sum, the appearance of cells with one *ori1* focus instead of two, elongation of cells without division for more than two generations, reduction in *ori1/ter1* ratio and loss of polar localization of *ori1* are all consistent with a block to elongation of Chr1 replication forks.

In newborn cells that showed the continued presence of a single *ori1* focus, the *ori2* focus also remained single during the time lapse experiment, indicating that Chr2 replication initiation depends on the prior replication of Chr1 (Fig 1B, panels 5–8). We were able to reverse the Chr1-replication block by removing the inducer of Tus expression (arabinose) using a microfluidic chamber (S1 Movie; S2 Fig). Upon removal of the inducer, *ori1* foci duplicated within ~30 min, followed by duplication of the *ori2* focus and resumption of cell division. The removal of arabinose also caused a significant decrease in Tus protein level, explaining the resumption of *ori1* duplication (S3A Fig). These results demonstrate that our approach to block Chr1 replication arrests the progression of the cell cycle (Chr2 replication and cell division) in a reversible fashion. Although it is possible that the cell division block could be responsible for blocking Chr2 replication, our results discussed later argue against this possibility.

We restricted our analysis of *ori2* foci only to cells that showed a single *ori1* focus, which accounted for ~50% of all cells. While majority of these cells had one *ori2* focus, some cells exhibited duplication of *ori2* foci within the duration of the time-lapse (Fig 1B, panels 9–12). On a population basis, by counting foci in cells 150 min after the induction of Chr1 block, 13% of one *ori1* cells were seen to have two or more *ori2* foci (Fig 1D), indicating that Chr2 has some propensity to initiate replication independent of Chr1 replication. Cells with one *ori1* and two *ori2* foci were longer than cells with one *ori1* and one *ori2* foci: 4.0 ± 0.3 μm as compared to 3.0 ± 0.2 μm (mean ± standard error, Fig 1E), suggesting that given enough accumulation of mass, Chr2 can replicate in the absence of Chr1 replication. This is unlikely to be due to increase of RctB concentration over time, which remains essentially unchanged in Chr1 replication-blocked cells (S3B Fig). Importantly, the results provide the initial indication that unreplicated *crtS* has activity, as we show later.

### A plasmid clone of *crtS* suffices for Chr2 initiation in Chr1 replication-blocked cells

**Fig 2:**
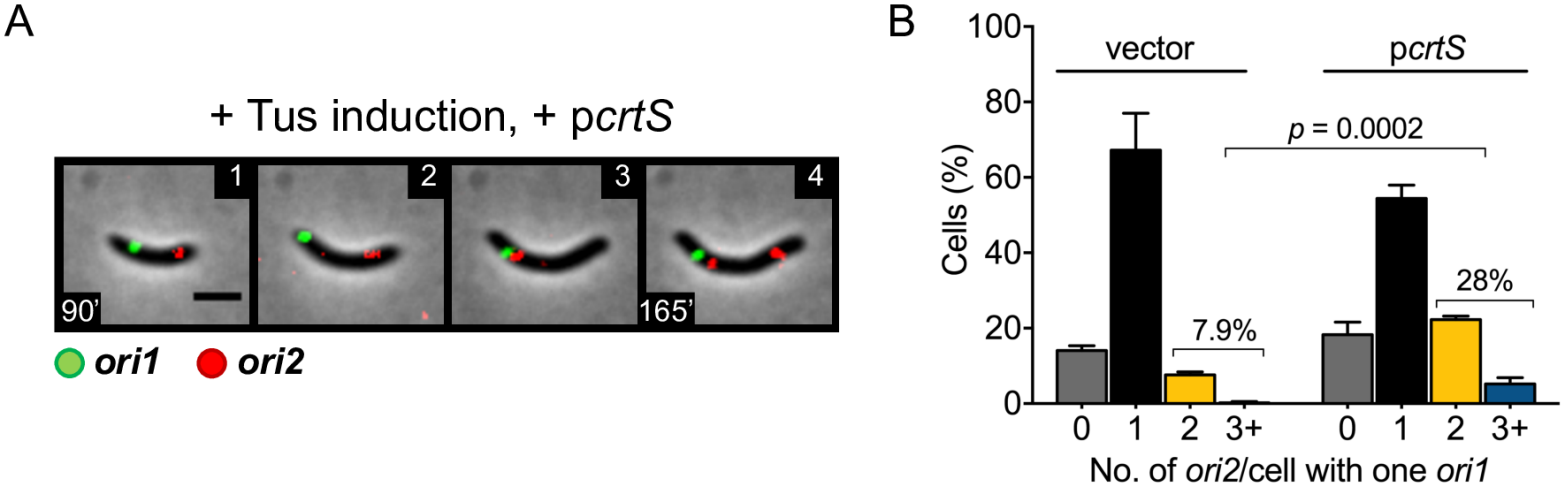
p*crtS* allows Chr2 replication in Chr1 replication-blocked cells. (A) Time-lapse microscopy of a cell with p*crtS* (CVC3028) grown in the presence of 0.2% arabinose. Under this condition, cells with two or more *ori2* foci can be seen in cells with one *ori1* focus (panel 4), indicating Chr2 replication in the absence of Chr1 replication. Scale bar, 2 μm. (B) Histogram of *ori2* foci number per cell (x-axis) as percentages of cells that have one *ori1* focus (y-axis). Note that the percentage of cells with two or more *ori2* foci (yellow + blue bars) increases from 7.9 ± 0.9% (Σn = 498 cells) in vector carrying cells (CVC3145) to 28 ± 2.1% (Σn = 590 cells) in p*crtS* carrying cells. Data represents mean ± SEM of percentages calculated from three biological replicates with at least 100 cells in each replicate. Statistical significance was calculated using a Student’s *t*-test.

An earlier study established that replication of the *crtS* locus on Chr1 is required for Chr2 replication [15]. We also find that the 153-bp *crtS* in a plasmid (p*crtS*) increases Chr2 replication (S4A Fig) [14]. To test whether the *crtS* is the only locus of Chr1 whose replication was required for licensing Chr2 replication, we supplied p*crtS*, in cells where Chr1 replication was blocked by the Tus-*ter* complex. In Chr1-replication blocked cells with p*crtS*, the number of cells with single *ori1* focus and two or more *ori2* foci increased 3-fold as compared to the empty vector (from 8% to 28%, counted after 150 min of Tus induction) (Fig 2). Under the growth condition used for microscopy, relative to *ori1* the copy number of p*crtS* was approximated by qPCR to be 2.5 ± 1.3 under Chr1-replication permissive growth and 4.5 ± 1.6 under Chr1-replication blocked growth. The presence of p*crtS* had no effect on Chr1 replication under Tus-*ter* block, as the distribution of *ori1* focus was similar whether the cells had p*crtS* or the empty vector (S5 Fig). The 153 bp *crtS* locus thus appears sufficient to license Chr2 replication without requiring replication of any other Chr1 locus.

### Two unreplicated *crtS* sites can license Chr2 replication

Our results so far are consistent with the view that *crtS* needs to be newly replicated for timely licensing of Chr2 replication. How does replication help? To test whether simply doubling the *crtS* copy number, that normally occurs as the replication fork crosses the site, could suffice for initiating Chr2 replication, an extra copy of *crtS* was inserted in Chr1 either 10 kb upstream of the native locus at 0.80 Mb or 900 kb downstream of the native locus at 1.84 Mb (hereafter called two-*crtS* cells). We also constructed a strain containing three copies of *crtS* that included both the ectopic copies in addition to the native copy. The strains with extra *crtS* copies were constructed with or without the Tus-*ter* block in Chr1. The cells without the Tus-*ter* block grew normally, as seen by their growth curves and average cell length (S6 Fig). We also deleted the native *crtS* locus from the strain where the added *crtS* copy was 10 kb upstream. Its growth rate, average cell length and number of *ori2* foci were similar to those of isogenic cells containing *crtS* at the native locus only, indicating that the added *crtS* functions normally at the new location.

**Fig 3:**
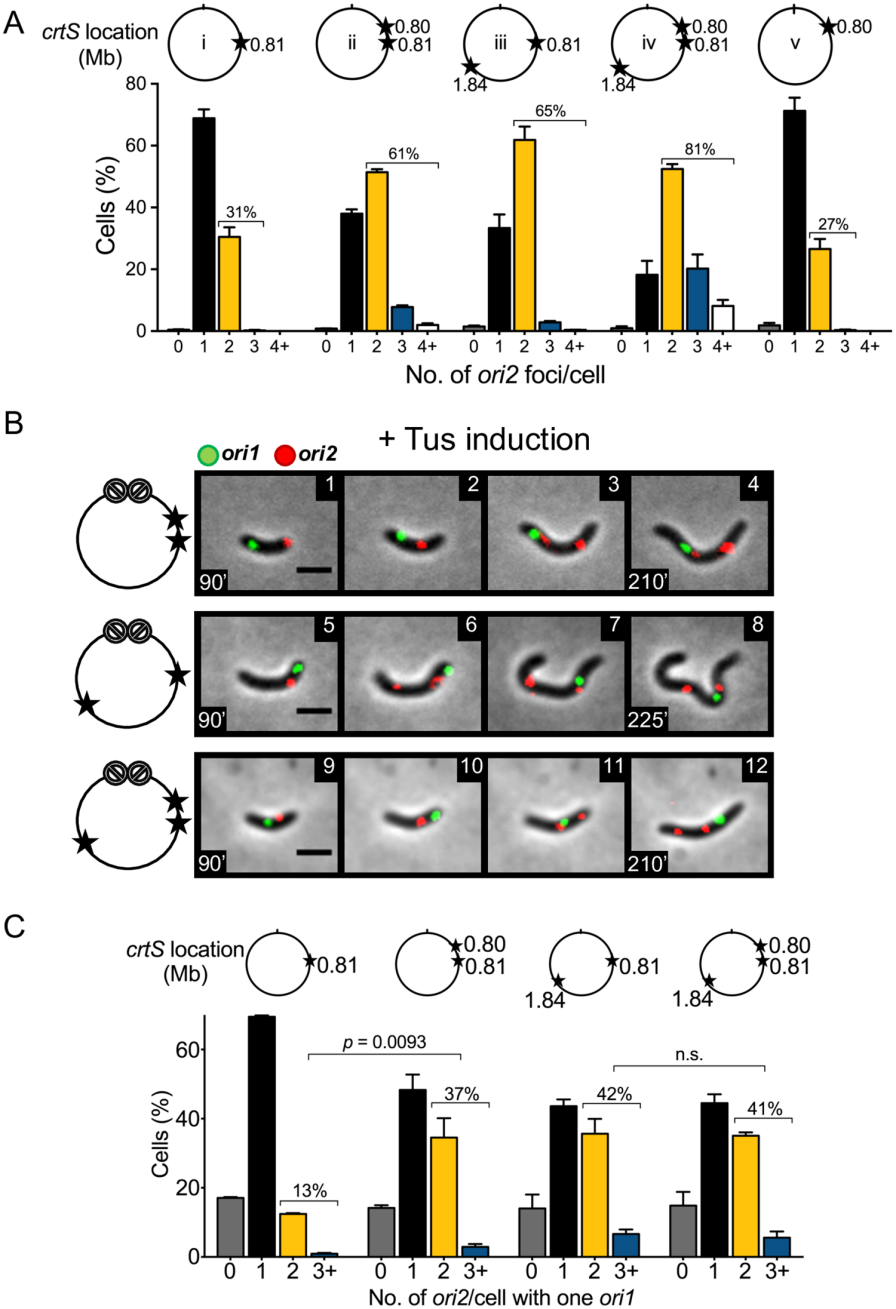
Extra copies of *crtS* in Chr1 promote Chr2 replication in Chr1 replication-blocked cells. (A) Histograms of *ori2* foci per cell in different strains when Chr1 replication was not blocked. The circles above the histograms show the location of *crtS* copies (stars) in Chr1 and their distance in Mb from *ori1* (tick mark at 12 o’clock). In these experiments, only *ori2* was fluorescently marked. The number of cells with ≥2 *ori2* foci increases in strains with two *crtS* copies (CVC3061 (ii) and CVC3093 (iii)), and increases even more in cells with three *crtS* copies (CVC3151 (iv)). Total cells (Σn) counted in (ii) to (iv) were 1233, 1609 and 1329, respectively. Deletion of the native *crtS* returns the distribution to that of WT cells (CVC3112 (v); Σn = 1346). (B) Time-lapse microscopy of cells with extra copies of *crtS* in Chr1 when its replication was blocked (CVC3056, panels 1–4; CVC3092, panels 5–8; and CVC3150, panels 9–12). In the schematics, the block to Chr1 replication is shown by crossed circles. In these cells, *ori2* foci duplicated frequently even as *ori1* focus remained unduplicated. Scale bars, 2 μm. (C) Histograms of *ori2* foci number per cell at 150 min after addition of 0.2% arabinose. Percentage of cells with one *ori1* focus and ≥2 *ori2* foci increases from 13 ± 0.15% (Σn = 522) in cells with one *crtS* (CVC3022) to 37 ± 5.1% (Σn = 726) in cells with an extra *crtS* 10 kb upstream (CVC3056), to 42 ± 5.6% (Σn = 516) in cells with an extra *crtS* 900 kb downstream (CVC3092), and to 41 ± 1.6% (Σn = 469) in cells with two extra *crtS* (CVC3151). The increase from two to three *crtS* carrying cells is not considered significant (*p*-value = 0.79). Data in (A) and (C) represent mean ± SEM of percentages calculated from three biological replicates with at least 100 cells in each replicate. Statistical significance was calculated using a Student’s *t*-test.

In two-*crtS* cells without the Tus-*ter* block, a larger percentage of cells contained two or more *ori2* foci (~60% in two-*crtS* cells vs. ~30% in one-*crtS* cells), indicating that Chr2 initiated earlier in the cell cycle than it did in one-*crtS* cells (Fig 3A). This was true whether the second *crtS* copy was close or far from the native site. The average length of cells with two *ori2* foci was also shorter in the presence of extra copies of *crtS*, indicating earlier initiation of Chr2 replication in the cell cycle (S6C Fig). The presence of three *crtS* copies further increased Chr2 replication such that nearly 80% of cells contained two or more *ori2* foci. These results are consistent with findings in an earlier study that showed that *crtS* activity is limiting for Chr2 replication under normal growth [15].

We next blocked replication of Chr1 by inducing Tus expression. Cells with one *ori1* focus showed two *ori2* foci in nearly 40% of two-*crtS* cells (Fig 3B and 3C), which is a 3-fold higher frequency than that was seen in one-*crtS* cells. Since Chr2 replication initiated more efficiently with two unreplicated copies of *crtS*, it would suggest that a role of replication is to double the *crtS* gene dosage and the act of replication may not be required for *crtS* function. In other words, an unreplicated copy of *crtS* has activity towards initiating Chr2 replication. The initiation was also similarly efficient whether the two *crtS* sites were near or far apart, suggesting that the sites probably do not interact with each other for function.

Since when Chr1 replication is not blocked, cells with more than one *crtS* copy also have more *ori2* foci, a concern can be that the increased number of cells with two *ori2* foci when Chr1 replication is blocked is a mere reflection of the *ori2* copy number in the replication-permissive mother cell rather than the consequence of new Chr2 initiation events in the absence of Chr1 replication. To address this concern, individual cells were followed by time-lapse microscopy at 20 min intervals beginning at 30 min after the addition of the inducer to block Chr1 replication (S7 Fig). Cells had either one, two or three *crtS* copies, as in Fig 3C. Cells were followed from the time a mother cell with two *ori1* foci divided into daughters with one *ori1* focus. Most of these cells were born with one *ori2* focus. During the course of time-lapse spanning ~ 150 min, the *ori2* focus duplicated earlier and in more cells when they had more than one copy of *crtS*. Only in about 4–9% of the newborn cells with multiple *crtS* copies, there were two *ori2* foci and one *ori1* focus. These *ori2* foci could have been inherited from the mother cell or be the product of duplication within the first 20 min interval of the time-lapse. These results indicate that in cells with two or more *ori2* foci scored at the end of time-lapse, the foci were mostly products of new duplication rather than inheritance from the mother (Fig 3C).

Increasing the number of *crtS* copies from two to three did not significantly enhance the number of cells with two or more *ori2* (Fig 3C, S7 Fig). In the absence of Chr1 replication, other factors necessary for replication probably become limiting for Chr2 initiation. We note that RctB levels tend to increase with the number of *crtS* copies (S8 Fig). This is possibly due to increased Chr2 replication and consequently higher dosage of Chr2-encoded *rctB* gene.

A block of Chr1 replication in two-*crtS* cells also blocked cell division as it did in one-*crtS* cells. Since in replication-blocked two-*crtS* cells Chr2 initiated efficiently, it is unlikely that the cell division block could be preventing Chr2 initiation (Fig 1B, panels 5–8).

### Increasing RctB supply obviates the need for Chr1 replication

The replication licensing activity of *crtS* is most probably mediated through RctB, so far the only factor known to bind *crtS* [14]. Moreover, increasing the supply of RctB increases Chr2 replication, suggesting that the initiator is normally limiting for replication [4, 6]. These considerations led us to ask whether increasing RctB can overcome the requirement of Chr1 replication for Chr2 replication.

**Fig 4:**
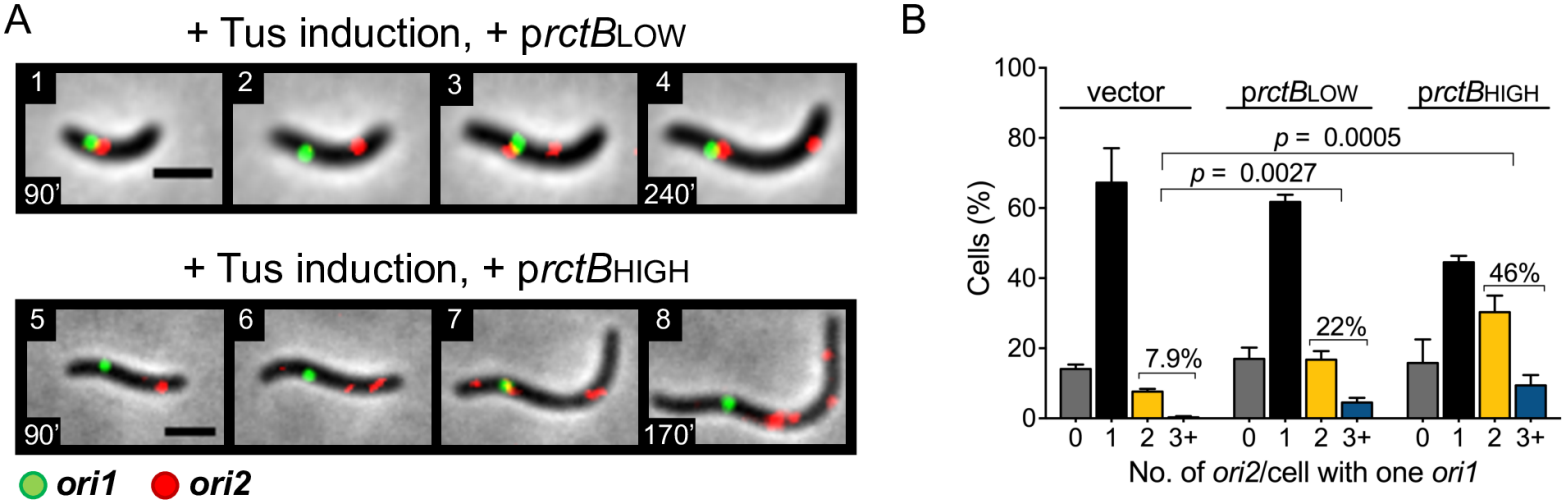
Increasing RctB concentration promotes Chr2 replication in Chr1 replication-blocked cells. (A) Time-lapse microscopy of Chr1 replication-blocked cells with additional RctB at low (CVC3052) or high (CVC3125) levels supplied from plasmid p*rctB*LOW or p*rctB*HIGH. Increasing RctB using either plasmid allowed Chr2 replication independent of Chr1 replication. Scale bars, 2 μm. (B) Histogram of *ori2* foci number per cell in the presence of low or high RctB, 150 min after addition of 0.2% arabinose. Percentage of cells with one *ori1* focus and ≥2 *ori2* foci (yellow + blue bar) increased from 7.9 ± 0.9% (Σn = 498) in cells with vector (CVC3145) to 22 ± 1.9% (Σn = 622) in cells with p*rctB*LOW (CVC3052) and to 46 ± 3.5% (Σn = 516) in cells with p*rctB*HIGH (CVC3125). Data represent mean ± SEM of percentages calculated from three biological replicates with at least 100 cells in each replicate. Statistical significance was calculated using a Student’s *t*-test.

RctB was supplied using plasmids that expressed the initiator constitutively. One plasmid produced RctB at near physiological level (p*rctB*LOW) and another at nearly a 9-fold higher level (p*rctB*HIGH), as quantified by Western blotting (S9A Fig). As expected, increasing *rctB* expression increased Chr2 replication in cells with p*rctB*HIGH when under Chr1 replication-permissive condition (S9B Fig). When Chr1 replication was blocked, a roughly 2-fold increase was observed in the number of cells with one *ori1* and two *ori2* foci in the presence of p*rctB*LOW as compared to the empty vector (from 8% to 22%, 150 min after Tus induction) (Fig 4A and 4B). In the presence of p*rctB*HIGH, the increase in the number of such cells was about 6-fold, accounting for roughly 50% of cells with one *ori1* focus (Fig 4B). Thus, increasing the supply of RctB can overcome the dependence on Chr1 replication. We believe that although normally two *crtS* copies are required, one *crtS* copy becomes adequate when the concentration of its interacting partner (RctB) is increased—a mass action effect. This rescue, we show later, depends on the presence of the solo unreplicated *crtS* copy, consistent with RctB being the target of *crtS*.

### *crtS* remodels RctB

RctB binding to *crtS* has been detected by DNase I footprinting and in cross-linking experiments [14]. The interaction of RctB with *crtS* likely remodels the protein and increases its initiator function, since in the presence of the site, RctB binding to 12- and 39-mers *in vivo* changes in a manner that should favor initiation. RctB is known to require remodeling to function since it depends on molecular chaperones, DnaK and DnaJ, for binding to both 12- and 39-mers and for maintaining a mini-Chr2 in *E*. *coli* [22]. The replication defect of mini-Chr2 in Δ*dnaKJ* cells could be partially overcome by providing p*crtS* [14]. These studies suggest that *crtS* has chaperone-like remodeling activity on RctB.

**Fig 5:**
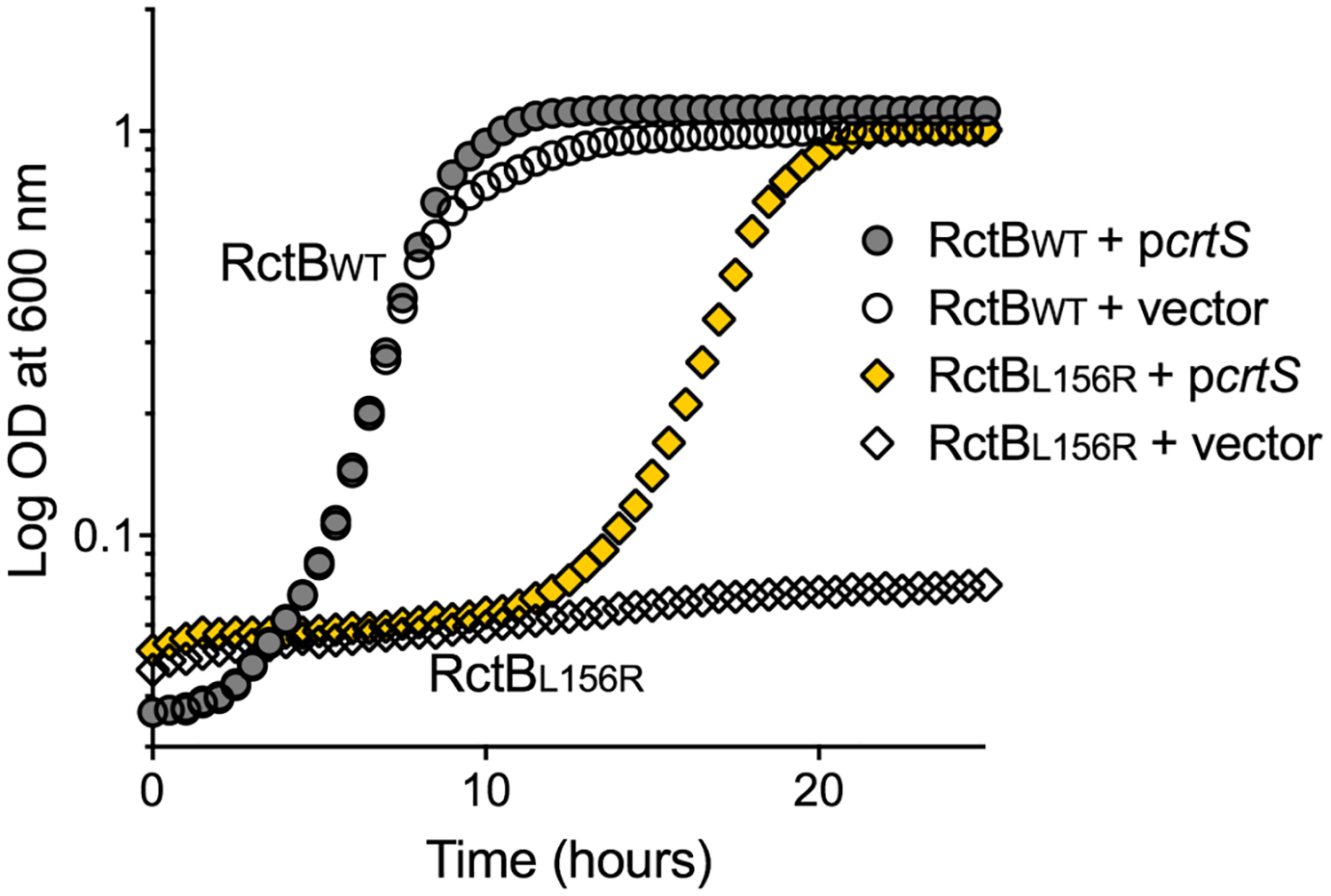
p*crtS* promotes growth of cells dependent on RctB function. Growth curve of *E*. *coli* cells after transformation with three plasmids: One is p*rctB* (circles, pTVC14) or p*rctB*L156R (diamonds, pJJ263); the second is p*crtS* (filled-in symbols, pBJH188) or the corresponding empty vector (empty symbols, pACYC177), and the third is mini-Chr2 (pJJ114). The results show that the DnaK-interaction defective mutant, RctBL156R, is able to support growth of *E*. *coli* dependent on mini-Chr2 replication, when provided with p*crtS*. Representative growth curves are shown from two biological replicates each performed in triplicates.

To further probe the chaperone-like function of *crtS*, we tested an RctB mutant (RctBL156R) that is significantly defective in interactions with DnaK [9]. This mutant binds to 12-mers poorly and does not support mini-Chr2 replication even in WT (*dnaKJ*^+^) *E*. *coli*. Cells dependent on mini-Chr2 replication grew when RctBL156R was the initiator, provided p*crtS* was also present (Fig 5). In comparison to the previous study using a host bearing a *dnaK* mutation, which can have pleiotropic effects, the present results obtained in the WT (*dnaK*^+^) background using a RctB mutant that is specifically defective in interactions with DnaK provide stronger support for the remodeling activity of *crtS*. Since RctBL156R retains some capacity to interact with DnaK, *crtS* could be helping RctB-DNA interaction, rather than substituting for the DnaK function. In either case, the results are consistent with a remodeling function of *crtS*. This is further supported by the observation that p*crtS* enhanced the growth of *E*. *coli* dependent on mini-Chr2 replication when the mini-Chr2 origin, rather than the initiator RctB, was defective due to insertion of 5 bp within it [11] (S10 Fig).

### *crtS* is required for Chr2 replication in each cell cycle

Deletion of *crtS* is detrimental for cell viability [15]. Survivors are found where either Chr1 and Chr2 have fused or mutations are present. The suppressor mutations so far have been found only in *rctB* or in its promoter. In view of this finding, we sequenced the whole genome of an apparently viable Δ*crtS* strain constructed in our earlier study [14]. The sequencing indeed revealed a mutation in *rctB* (S11A Fig). Thus, the presence of a compensatory mutation or genomic rearrangements seems necessary for viability of Δ*crtS* strains. The RctB mutant was still responsive to *crtS*, since the initiator activity of the mutant increased in the presence of *crtS* (S11B Fig). This implies that the mutant is gained in functions not controlled by *crtS*.

**Fig 6:**
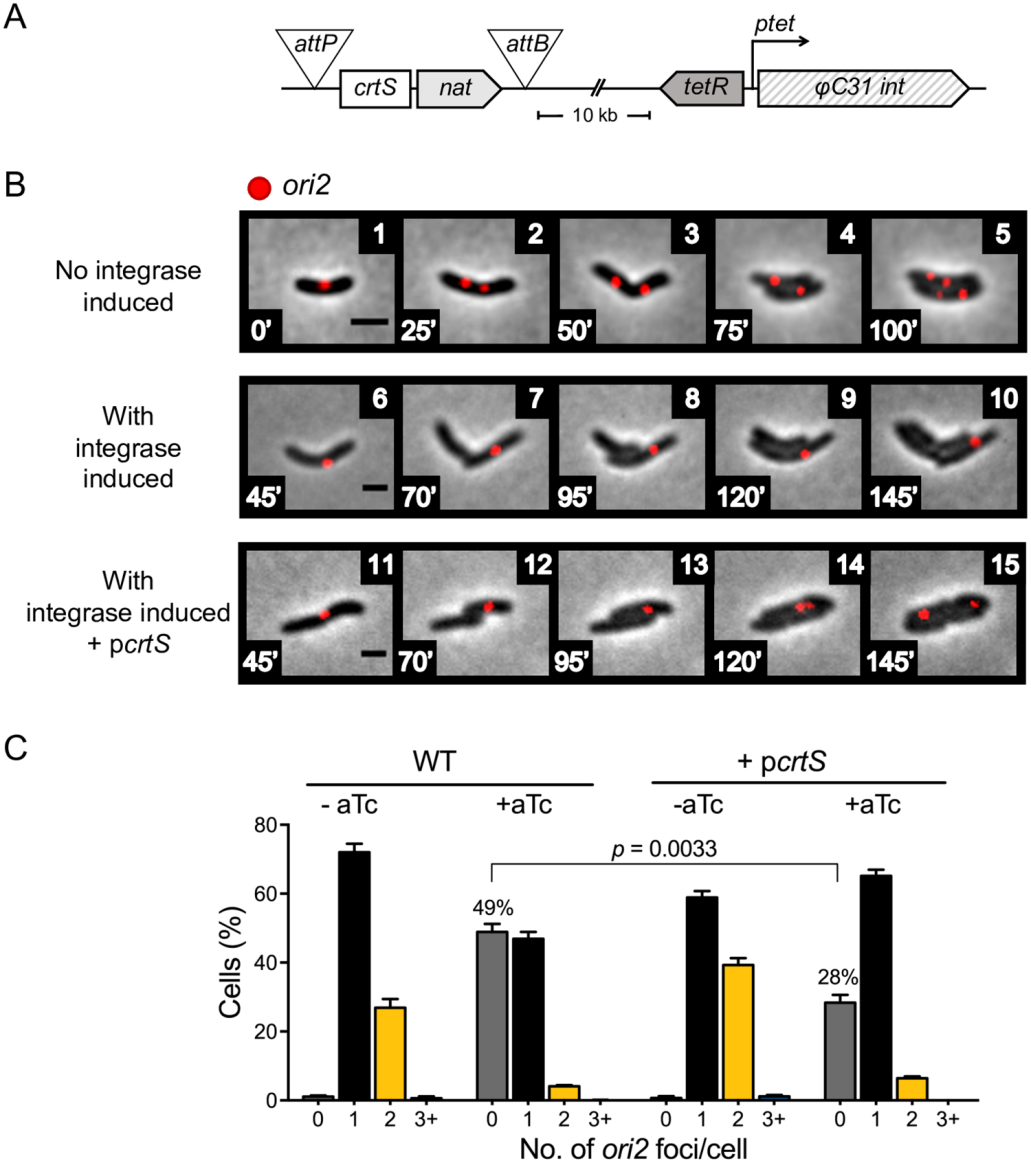
Cessation of Chr2 replication upon excision of *crtS*. (A) Scheme to excise *crtS* by flanking it with *attB* and *attP* sites and inducing the ΦC31 integrase gene from the *ptet* promoter with the inducer, aTc. Without induction, *ptet* is repressed by the product of *tetR*. The *nat* marker allows to monitor the frequency of excision. (B) Time-lapse microscopy of cells (CVC3082) without the integrase induction (panels 1–5), the same cells with induction (panels 6–10), and when the induction was in p*crtS*-carrying cells (CVC3137, panels 11–15). Chr2 replicates normally in the absence of the inducer, as seen by the increase from one to four *ori2* (red foci) per cell in about two generations. Induction of the integrase shows the inability of Chr2 to replicate even as cells continue to grow and divide, since only one *ori2* focus is visible after two generations. *ori2* foci duplication is seen upon integrase induction when p*crtS* was present. Scale bars, 2 μm. (C) Histogram of cells with different numbers of *ori2* foci in cells with or without p*crtS* after 150 min of integrase induction. Upon induction, the number of cells with no *ori2* focus increased significantly. The presence of p*crtS* partially complements the Chr2 replication defect upon excision of the chromosomal copy of *crtS*, as seen by the reduction in cells with no *ori2* focus from 49 ± 2.3% in WT cells to 28 ± 2.3% in p*crtS*-carrying cells. Total cells (Σn) counted for: WT -aTc = 1340, WT +aTc = 1250; p*crtS* -aTc = 1097, p*crtS* + aTc = 1173.

To study the consequences of *crtS* deletion on a shorter time scale, we developed a method to excise *crtS* and study its effect on Chr2 replication in real-time. Native *crtS* was flanked with the *attB* and *attP* attachment sites of ΦC31 phage and the cognate integrase gene was placed under the control of the *ptet* promoter [23, 24] (Fig 6A). Upon induction of the integrase by anhydrotetracycline (aTc), cells continued to divide but duplication of the *ori2* foci ceased, giving rise to daughter cells that lacked any *ori2* focus (Fig 6B, S12 Fig and S2 Movie). The continuation of cell division confirms a prior observation that the cell cycle is insensitive to block in Chr2 replication [17]. On a population basis, after about two generations of adding aTc, ~50% of cells did not show an *ori2* focus. The proportion of cells without *ori2* reduced to ~30% in the presence of p*crtS* (Fig 6C), implying that the presence of the plasmid helped Chr2 replication. The inability of Chr2 to replicate upon induction of *crtS* excision indicates that for licensing *crtS* needs to be replicated anew in each cell cycle.

### *crtS* is required to promote Chr2 replication in the presence of excess RctB

The ability to follow the consequences of *crtS* excision in real-time allowed us to test whether a single unreplicated copy of *crtS* has activity towards RctB. Since *crtS* is crucial for Chr2 replication under normal conditions, an unreplicated *crtS* copy might still be required for Chr2 replication that is promoted by supplying excess RctB in Chr1 replication-blocked cells (Fig 4).

**Fig 7:**
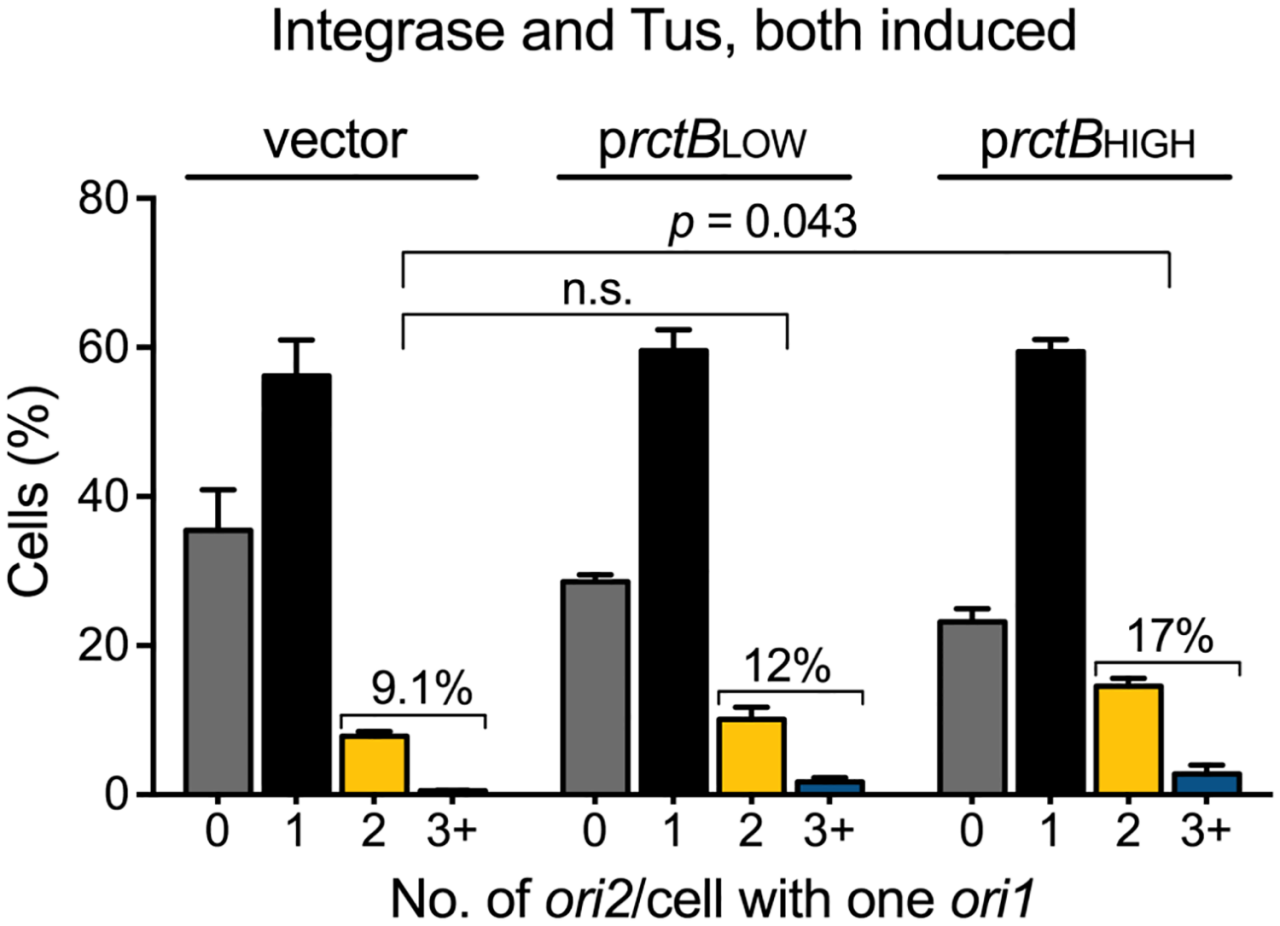
Excess RctB requires the unreplicated *crtS* site to promote Chr2 replication. Histogram of *ori2* foci numbers in cells with either the vector (CVC3169), or p*rctB*LOW (CVC3167) or p*rctB*HIGH (CVC3168), after integrase and Tus induction to excise *crtS* and block Chr1 replication. The number of cells with two *ori2* foci does not increase significantly in the presence of the vector vs. p*rctB*LOW (9.1 ± 0.5%, Σn = 544 vs. 12 ± 2.1%, Σn = 456), and increases about 2-fold to 17 ± 2.1% (Σn = 551) in the presence of p*rctB*HIGH. Note that when *crtS* was not excised the increases were higher, about 2- and 5-fold in the presence of p*rctB*LOW and p*rctB*HIGH, respectively (Fig 4B). Data represents mean ± SEM of percentages calculated from three biological replicates with at least 100 cells in each replicate. Statistical significance was calculated using a Student’s *t*-test.

To test for the *crtS* dependence, we excised the site using the ΦC31 system as before. One hour after inducing *crtS* excision, we induced Tus expression to block Chr1 replication. When *crtS* was not excised, cells containing duplicated *ori2* were 2-fold more in the presence of p*rctB*LOW and 5-fold more in the presence of p*rctB*HIGH compared to cells containing the empty vector (Fig 4B). In contrast, when *crtS* was excised, the percentage of cells with duplicated *ori2* was similar in cells containing the empty vector or p*rctB*LOW (9% vs. 12%) and only 2-fold more in cells containing p*rctB*HIGH (9% vs. 17%) (Fig 7). The residual replication of Chr2 upon *crtS* deletion could be due to some spontaneous remodeling. These results are consistent with the view that unreplicated *crtS* possesses significant activity towards promoting RctB function, which becomes particularly crucial near the physiological concentrations of the initiator.

## Discussion

In eukaryotic cells, the norm is to have more than one chromosome. That this can be true in some bacteria elicited interest in studying mechanisms of chromosome maintenance that evolved in bacteria and their relation to those operating in eukaryotes. With only two chromosomes, Chr1 and Chr2, *V*. *cholerae* has proven to be the most tractable organism to address these questions. Replication timing of the two chromosomes, however, is found to be coordinated by an unprecedented mechanism. The timing of Chr2 replication initiation depends on prior duplication of a site, *crtS*, in Chr1. Chr2 initiation depends not only on the timing of Chr1 initiation but also on the time taken by the Chr1 replication fork to travel to the *crtS* locus [15]. The position of *crtS* in Chr1 thus dictates the timing of its function, exemplifying the importance of chromosomal location of a gene for its function, a topic that is receiving increasing attention [25].

Here we have addressed the role of replication in *crtS* function and find that doubling of *crtS* site dosage (without requiring the act of replication) suffices for function. The finding that *crtS* need not be newly duplicated to be active should facilitate determining the mechanistic underpinnings of its function in purified systems.

### Role of replication in *crtS* function

There are several ways the passage of a replication fork can influence the activity of a site. Simply doubling the copy number of a site can increase the cumulative activity that may suffice for function. Indeed, our present data are consistent with this possibility since two unreplicated copies of *crtS* promoted Chr2 replication more effectively than one unreplicated copy (Fig 3).

Our finding that the act of replication is not essential also makes the following models less likely, though not mutually exclusive. 1. Replication changes *crtS* topology: The passage of a replication fork can drastically change DNA topology in its wake. An approaching fork introduces positive supercoils, whereas after passage of the fork, the DNA gets relaxed or negatively supercoiled [26]. The template strand, copied by lagging strand synthesis, also briefly remains single-stranded at the growing fork. DNA topology-sensitive DNA-protein interactions thus can be profoundly altered during and immediately after the passage of the fork. Our results indicate that the above DNA topology changes are not obligatory for *crtS* function. 2. Replication allows sandwiching of the target: It is conceivable that *crtS* function may require at least two copies of the site, e.g., if the function involves sandwiching the target. If interactions between sisters were a requirement, it occurred in our replication-blocked experiments without hemi-catenation [26], and without hemi-methylation and SeqA binding [27], common in newly replicated sister chromosomes, and with nearly equal facility when two unreplicated copies were near or far from each other (Fig 3). 3. Replication clears *crtS* of bound factors: It is possible that the passage of the fork dissociates factors that may bind and inhibit *crtS* function [28].

We note that Chr2 does not replicate in all cells in the absence of Chr1 replication, even when provided with two *crtS* copies. A likely reason could be blocking of Chr1-replication. Since most, if not all, required factors for replication are synthesized from Chr1, they may have been inadequately produced when cells had a single copy of Chr1. This is indicated when *crtS* copies were increased from two to three. Cells with 2 or more *ori2* increased from 61% to 81% under replication permissive condition (Fig 3A), but remain unchanged at ~40% under replication blocked condition, despite the extra *crtS* (Fig 3C). Unless the cause for Chr2 not replicating in all cells is better understood, whether replication does more than just double the *crtS* copy number, such as change DNA topology as discussed earlier, cannot be ruled out from the present results.

### *crtS* serves only a positive role in Chr2 replication

**Fig 8:**
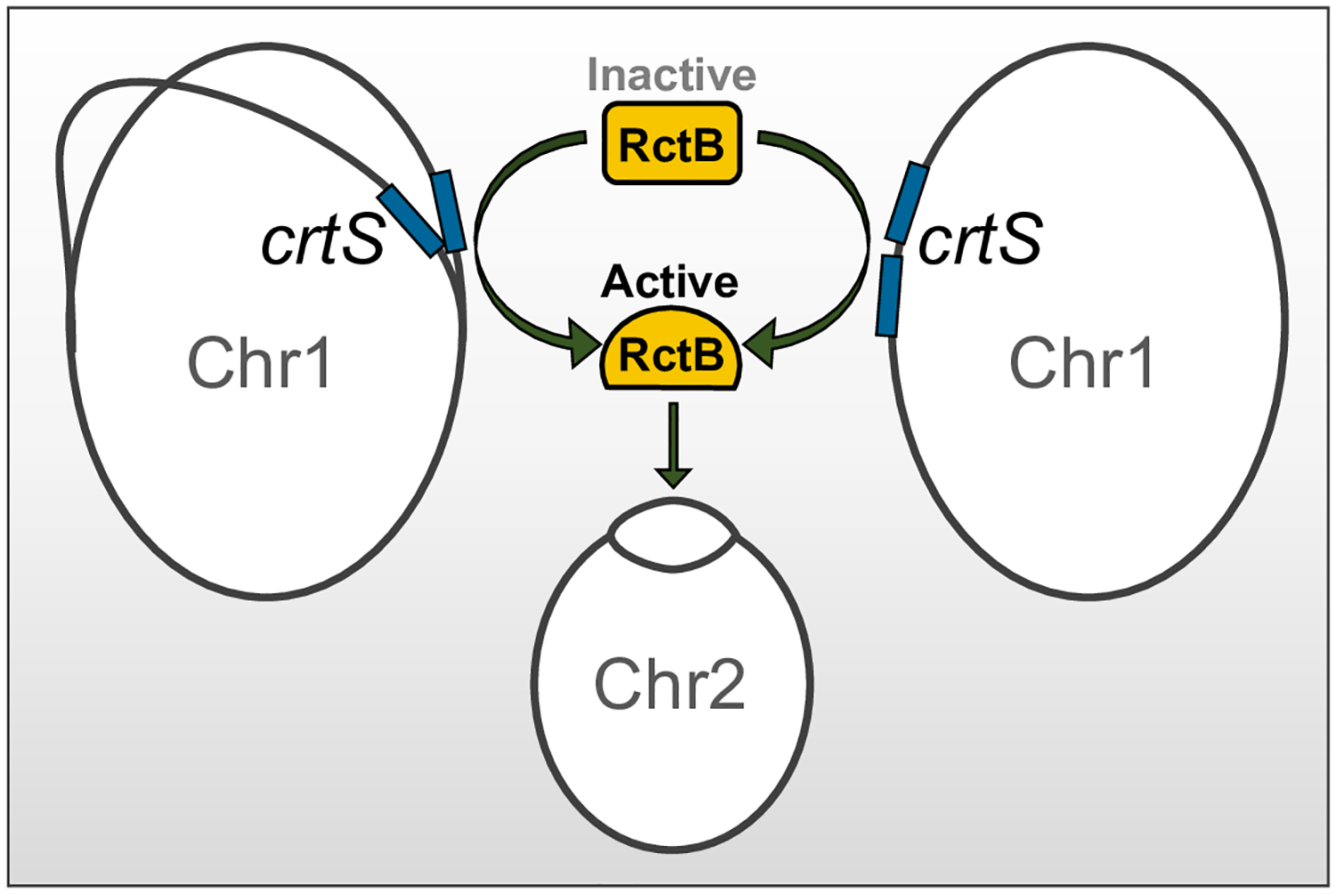
Model for Chr2 replication licensing by remodeled RctB. The triggering of Chr2 replication initiation requires two copies of *crtS*. The two copies are normally produced by passage of the Chr1 replication fork across the *crtS* site (left diagram) or, can also be provided in the absence of Chr1 replication by inserting an extra copy of *crtS* (right diagram). This suggests that the product of replication but not the passage of the fork *per se* is obligatory for *crtS* function. In our experiments, the requirement for a second copy of *crtS* could be obviated by increasing RctB supply (Fig 4). This, together with the findings that to date all revertants of Δ*crtS* map in *rctB* and that *crtS* alters the DNA binding activities of RctB, lead us to propose that *crtS* activates RctB. The two *crtS* copies are apparently required to activate RctB sufficiently for initiation.

It is possible that unreplicated *crtS* could be an inhibitor of Chr2 replication, for example, by titrating an activator of Chr2 replication or by coupling with *ori2*, as in plasmid handcuffing [29]. In these scenarios, inhibitory activity is expected to increase in the presence of additional unreplicated *crtS* copies. Our finding that unreplicated *crtS* copies instead increase Chr2 replication makes these scenarios unlikely. Moreover, should *crtS* be functioning only as an inhibitor in the absence of replication, deletion of the site should suffice to activate Chr2 replication, but it does not. In sum, *crtS* appears to serve only a positive role in *ori2* firing. A single copy of *crtS* has significant but insufficient activity. Doubling its copy number, normally afforded by replication, appears to raise the activity above the threshold required for initiating replication (Fig 8).

### *crtS* remodels RctB

We showed earlier that RctB binding characteristics change in the presence of *crtS* [14] and provide new evidence here that the site relieves the chaperone requirement of RctB activity to some extent and allows the initiator to function with a defective origin (Fig 5 and S10 Fig). These results are consistent with *crtS* being a remodeler of RctB that promotes its initiator function, analogous to the role played by chaperones [9]. There is precedence for DNA-binding mediated remodeling of initiator proteins and modulation of their function by chaperones [30–36]. In our experiments, excess RctB could relieve the requirement for *crtS* replication, but the presence of an unreplicated *crtS* copy was still helpful (Figs 4 vs. 7). Even though two *crtS* copies are required, presumably at the increased RctB concentration, there was enough interactions with an unreplicated copy of *crtS* to yield sufficient remodeled RctB for initiating Chr2 replication. There is not sufficient structural information available on RctB at present for a meaningful discussion on the molecular nature of the remodeling.

### Once per cell-cycle control of Chr2 replication

How does a cell ensure once per cell cycle replication of Chr2? By linking the replication of Chr2 to that of Chr1 via *crtS* the cell ensures that a trigger is received by Chr2 every time Chr1 is replicated. Given that Chr1 initiation is tightly regulated, this is a fail proof mechanism for licensing Chr2 replication. Our results suggest that doubling the *crtS* copy number could serve as the sensor for Chr2 initiation, by affecting the binding preference of RctB. However, the nature of the block that prevents Chr2 replication in the absence of *crtS* duplication is not known (Fig 1), making it difficult to speculate how the block is overcome efficiently upon *crtS* duplication (Fig 3). The Chr2 initiator RctB is limiting for Chr2 replication and indications are strong that *crtS* helps to overcome this rate-limiting component. The negative regulatory forces then prevent further replication in the same cell cycle to ensure only one round of replication per cell cycle.

### Activity of chromosomal vs. plasmid-borne *crtS*

In our experiments, p*crtS* failed to fully complement excision of the chromosomal *crtS*, although the site was present in similar copy number when plasmid-borne (Figs 2B vs. 3C). What compromises *crtS* activity when plasmid-borne is not clear but p*crtS* is known to reduce colony size [14]. We report here further evidence for stress to cells by p*crtS* (S4B Fig). In the classical N16961 El Tor background of *V*. *cholerae*, colonies are smaller and display a rugose morphology in the presence of p*crtS* but not the vector. Although our microscopy strains do not show the rugose morphology (expected because they are *hapR*+ [37]), their colony sizes are still smaller. How p*crtS* affects global cell physiology remains unknown. Moreover, for unknown reasons, plasmids in general have reduced copy number and are unstable in *V*. *cholerae* [6]. The instability of p*crtS* and the stress caused by it are confounding factors in comparing *crtS* activity from the chromosome and the plasmid. Further studies on why plasmid copy number is lower in *V*. *cholerae* and how p*crtS* stresses cells will be required before the importance of location for *crtS* function can be understood.

### Origin licensing in eukaryotes and other bacteria

Origin licensing is a key mechanism for limiting initiation of eukaryotic DNA replication to once per cell cycle [38]. Although initiation occurs in S-phase, the helicases are loaded in the G1-phase and they cannot be loaded later in S-phase. A helicase-loaded origin in G1-phase is considered licensed to initiate replication, although the actual initiation awaits activation of the helicases in S-phase by kinases CDK and DDK.

It is clear that *crtS* participation is critical for origin firing of Chr2, but the exact step(s) of the initiation reaction in which *crtS* participation is required remains unclear. The initiators of the iteron family of plasmids believed to be the progenitor of Chr2 are known to facilitate helicase loading in addition to DNA binding [39, 40]. A *crtS*-remodeled initiator could also be more proficient in helicase loading, in which case it would have a role beyond formation of the initial initiator-origin complex. In phage λ, loading and activation of the helicase occur at separate stages, the latter requiring molecular chaperones [41]. A role of the chaperone-mimic *crtS* in loading/activation of the helicase therefore cannot be ruled out. In that case, the *crtS*-mediated licensing could be likened to the eukaryotic licensing of replication.

Among the members of the *Vibrionaceae* family, the sequences of *crtS* and *rctB* are conserved, making it likely that the origin-licensing system described in *V*. *cholerae* will apply to the entire *Vibrionaceae* family. Bacteria with multiple chromosomes are present in different classes of proteobacteria and probably evolved independently [42]. Of special interest is the ß-proteobacterium *Burkholderia cenocepacia* that possesses three chromosomes, c1, c2 and c3 [43]. c1 and c2 are analogous to the *V*. *cholerae* Chr1 and Chr2. The timing of replication of both c1 and c2 are fixed in the cell cycle, with c1 initiating first, whereas the timing of replication of c3 (which resembles plasmids) is more random, which is the norm for plasmids [44]. Examples of non-random timing of replication initiation also exists in the α-proteobacterium, *Sinorhizobium meliloti*, which possesses a chromosome and two megaplasmids that are replicated and segregated in a sequential manner [45], and *Brucella abortus*, whose Chr1 initiates replication before its Chr2 [46]. Moving forward, it will be important to determine how far the *Vibrio* paradigm applies to other bacteria possessing divided genomes.

## Materials and methods

### Strains and growth conditions

Bacterial strains, plasmids and primers are listed in S1–S3 Tables, respectively. Cloned regions were confirmed by sequencing. *E*. *coli* DH5Δ*lac* (BR2846) and Top10 (Invitrogen, Carlsbad, CA) were used for standard plasmid manipulations and propagation. *V*. *cholerae* strains used were derivatives of CVC1121, a *hapR*^+^ derivative of the sequenced strain N16961. *E*. *coli* strains were grown at 37 °C and *V*. *cholerae* at 30 °C. Antibiotics and their concentrations used in *E*. *coli* were: ampicillin 100 μg/ml, chloramphenicol 25 μg/ml, kanamycin 25 μg/ml, nourseothricin (Nat) (Gold Biotechnology, St. Louis, MO) 25 μg/ml, spectinomycin 50 μg/ml and zeocin (Invitrogen) 25 μg/ml. In *V*. *cholerae*, antibiotic concentrations used were the same except that chloramphenicol was used at 5 μg/ml and kanamycin at 12.5 μg/ml when cultures were grown in supplemented 1XM63 medium (1X M63 buffer, 1 mM CaCl_2_, 1 mM MgSO_4_, 0.001% vitamin B1, 0.2% fructose and 0.1% casamino acids) for microscopy. Inducers were used at the following concentrations when desired: IPTG at 100 μM, arabinose at 0.2%, and anhydrotetracycline (aTc) at 20 ng/ml.

### Natural transformation

This was used for most genetic manipulations of the *V*. *cholerae* chromosomes [47]. Cells were grown to log phase in LB medium at 30 °C and 1 ml of culture was washed twice in 1X M9 medium supplemented with 32 mM MgCl_2_ and 5 mM CaCl_2_. The washed cells were incubated with autoclaved chitin flakes (Sigma-Aldrich, St. Louis, MO) for 24 h at 30 °C without shaking. About 200 ng of linear DNA fragments (generated by either PCR or restriction digestion) with ~1 kb homology region at each flank were added to the cells and gently mixed, and the mixture was incubated for 24 h at 30 °C. The chitin flakes were vortexed to facilitate detachment of cells from the chitin surface and plated to select for transformants. The genetic alterations were confirmed by PCR, using primers flanking the altered regions.

### Expression of GFP, tdTomato and Tus proteins

The plasmid pRR06, containing *araC*, *pbad*-*tus*, *lacI*^q^, *ptac*-(*tdTomato*-pMT*parB gfp*-P1*parB*) and the *cat* cassette, was constructed using the Gibson Assembly kit (New England Biolabs, MA). The *tdTomato* gene was amplified from pALA2486 using primers RR53 and RR40, and pMT*parB* gene from pALA2819 using primers RR51 and RR41. The two genes were fused in frame and inserted between *ptac* and *gfp*-P1*parB* in pALA2705 replacing the *mCherry-pMTparB* to yield plasmid pRR01. The 6 kb *lacI*^q^ *ptac*-(*tdTomato*-pMT*parB gfp*-P1*parB t1t2*) from pRR01 was amplified using primers RR07 and RR29. The *araC pbad*-*tus* fragment was amplified from plasmid pBAD30-*tus* using primers RR05 and RR06, the *cat* gene from pACYC184 using primers RR09 and RR10, the 1 kb upstream and downstream flanking regions of *lacZ* from genomic DNA of CVC1121 using primers RR03 and RR04, and RR11 and RR12, respectively. Using the Gibson kit, the above fragments were assembled in the sequence: upstream *lacZ* flank, *araC pbad-tus*, *lacI*^q^ *ptac*-(*tdTomato*-pMT*parB gfp*-P1*parB t1t2*), *cat*, and downstream *lacZ* flank in pGEM-T-easy vector (Promega, WI). The ~12 kb region was excised using Not1 and used in natural transformation of CVC1121. Following selection on chloramphenicol plates, the resulting strain (CVC3001) was confirmed for Tus expression under arabinose control by Western blotting, and for tdTomato-pMTParB and GFP-P1ParB expression under IPTG control by fluorescence microscopy.

### Marking chromosomal origins with *parS* tags

P1*parS* Kn (at +135 kb on Chr1) and pMT*parS* Sp (at +40 kb on Chr2) were inserted in CVC1121 by natural transformation with genomic DNA from CVC1146 [17]. While the resulting strain CVC3009 showed the GFP-P1ParB (*ori1*) focus in all cells, it did not show tdTomato-pMTParB (*ori2*) focus in ~20% of the cells possibly due to slow maturation of tdTomato. This discouraged us from using tdTomato except when simultaneous monitoring of the two colors was necessary (such as time-lapse microscopy of Chr1-blocked cells). In situations where a more accurate count of *ori2* foci was desired, such as in Figs 3A, 6B and 6C, S4 and S9B Figs, GFP was used to mark *ori2* instead of tdTomato and *ori1* was not marked. In these cases, P1*parS*-Kn was inserted at +40 kb from *ori2* by natural transformation of strain CVC3001 with genomic DNA from CVC1166 that resulted in strain CVC3058.

### Blocking replication elongation from *ori1* by Tus-*ter* complex

The *E*. *coli ter* sites (23 bp) when properly oriented and bound by Tus protein serve as roadblocks to replication fork progression [18]. *E*. *coli ter* sites were inserted on either side of *ori1* in its active orientation, and the *E*. *coli* Tus protein was expressed from *pbad*. The *ter* sites were inserted by amplifying the *ori1* region of plasmid pGD101 with primers (RP017 and RP018) that added the 23 bp *ter* sites. In pGD101, *ori1* is linked to a *zeo* cassette that allows selection of transformants. A 1 kb region from each flank of *ori1* was amplified from *gidA* and *mioC* genes to provide homology for recombination in natural transformation experiments. The three fragments, *gidA-terB*, *terB-ori1-zeo-terB* and *terB-mioC* were joined in order by overlap extension PCR and the product was transferred to the chromosome by natural transformation. The resulting strain, CVC1157, proved to be inefficient in blocking replication forks of Chr1, necessitating addition of a second *ter* site in tandem to either flanks of *ori1*. The *terB*-flanked *ori1* region of CVC1157 was amplified in three ~1 kb segments as before using primers that added the 23 bp *terC* sites: RR66 and RR72 for *gidA-terC*, RR78 and RR79 for *terC-terB-ori1-terB-terC*, and RR80 and RR71 for *terC-mioC*. The three fragments were joined in an order to the pGEM-T-easy vector using the Gibson kit that resulted in pRR08. The assembled region (*gidA terC terB ori zeo terB terC mioC*, about 3 kb) was amplified from pRR08 and the product used in the natural transformation of CVC3009 that generated CVC3019. The Kn cassette of P1*parS* Kn was excised by supplying the Flp recombinase from pBRFLP as the cassette was flanked with FRT sites. The resulting Kn-sensitive derivative of CVC3019, CVC3022, was used in Fig 1.

### Measurement of p*crtS* copy number by qPCR

The copy number was approximated by measuring the relative marker frequency of the kanamycin resistance gene, *kanR2*, present in p*crtS*, to *ori1* on the chromosome. An aliquot of cell culture grown similarly as for time-lapse microscopy was diluted 1:100 in H_2_O, heated at 95 °C for 10 mins and frozen at -20 °C prior to being used directly as template for qPCR, which was performed as described previously [12]. The ratios were normalized to the ratio obtained from the DNA of CVC3013, which contains a *kanR2* inserted into *lacZ*, thus possessing a *kanR2/ori1* marker frequency ratio of ~1. Data represents mean ± standard deviation and was averaged from two biological replicates analyzed in duplicate.

### Insertion of extra *crtS* copies in Chr1

A copy of *crtS* was inserted 10 kb upstream of the native *crtS* locus in the intergenic region of VC0754 and VC0755. The upstream 1 kb (805058 to 805991) and downstream 1 kb (806014 to 83000) regions of the insertion site, and *crtS* were amplified from N16961 genomic DNA, and the *nat1* gene conferring nourseothricin resistance was amplified from pAM101 [48]. The amplified DNA fragments were assembled using the Gibson kit into pGEM-T-easy vector in the following order: Upstream 1 kb region, *crtS*, *nat1* and downstream 1 kb region. The assembled region (~ 3.3 kb) of the resulting plasmid, pRR16, was isolated by Not1 digestion and the fragment was used in natural transformation of CVC3022 and CVC3058 to generate strains CVC3056 and CVC3061, respectively, upon selection on nourseothricin plates. A copy of *crtS* was also inserted 900 kb downstream of the native *crtS* locus in the intergenic region of VC1710 and VC1711 at 1.84 Mb in Chr1. The regions flanking the insertion site were already present in pPS64. The *crtS* linked to *nat1* present in pRR16 was cloned into the PstI site of pPS64, resulting in pRR20. A Not1 fragment of pRR20 was used in natural transformation of CVC3022 and CVC3058 to generate CVC3092 and CVC3093, respectively. To insert a third *crtS*, the *nat1* gene was replaced with a Kn cassette in pRR16, and the Not1 fragment from the resultant plasmid, pRR25, was used in natural transformation of CVC3092 and CV3093 to generate strains CVC3150 and CVC3151, respectively, by selection on Kan plates.

### Deletion of the native *crtS* copy from a two-*crtS* strain

Plasmid pBJH245 contains the natural flanks of *crtS* and a *zeo* cassette in place of *crtS* [14]. The *zeo* cassette and the flanks were amplified from pBJH245. This product was used in natural transformation of CVC3061 to generate CVC3112 with the *zeo* cassette replacing the native *crtS* locus at 0.81 Mb and retaining only the *crtS* copy 10 kb upstream of the native locus.

### Excision of *crtS* by an inducible system

*crtS* was excised in real-time using the site-specific recombination system of ΦC31 phage. *crtS* was flanked with ΦC31 *attP* and *attB* sites, and the ΦC31 *int* gene was expressed from an aTc (IBA Lifesciences, Germany) inducible promoter, *ptet*. To monitor the loss of *crtS*, the gene was linked to a *nat* cassette [48]. The *crtS-nat* region was amplified from pRR16. The *attP* and *attB* sites were amplified separately from pTF6. pTF6 contains 50 bp *attB* and *attP* sites flanking the origin of pACYC184 and was constructed by inserting an *attP* p15A*ori attB* double-stranded IDT geneblock ligated into pACYC184 digested with SacII and XbaI. The cloning was done using the Gibson kit. The *attP* and *attB* sequences used were AGTAGTGCCCCAACTGGGGTAACCTTTGAGTTCTCTCAGTTGGGGGCGTA and CCGCGGTGCGGGTGCCAGGGCGTGCCCTTGGGCTCCCCGGGCGCGTACTCC, respectively. The natural flanks of *crtS* and the vector backbone were amplified from pBJH245. The amplified fragment (left-flank-of-*crtS* vector right-flank-of-*crtS*) was combined with *attP*, *crtS-nat* and attB fragments using the Gibson kit that generated pRR17 (which is same as pBJH245 except for the presence of *attP*-*crtS*-*nat*-*attB* in place of the *zeo* cassette). A linear 3.3 kb Not1 digested fragment that contained *crtS* and the flanking regions but not the vector sequences was used in natural transformation of CVC3058 to generate CVC3068 upon selection on nourseothricin plates. To supply the ΦC31 integrase under the control of *ptet* promoter, plasmid pRR21 was constructed which carried in order *bla*, *tetR* and *ptet*- ΦC31*int* genes in place of *crtS* of pRR16. The homology regions present in pRR16 allowed insertion of the elements 10 kb upstream of the native *crtS* locus. The *int* gene was obtained from plasmid pInt (Addgene plasmid #18941) [49] and was cloned downstream of *ptet* of plasmid pASK-IBA32 (IBA Life Sciences, Germany). The latter plasmid was also the source of *bla* and *tetR*. The *bla* gene allowed selection of transformants. The start codon of the *int* gene was changed from ATG to TTG to reduce the uninduced level of Int expression. The elements were assembled into a pGEM-T-easy vector by the Gibson kit that resulted in pRR21. A 4 kb Not1 fragment of pRR21 was used in the natural transformation of CVC3068 to generate CVC3082 upon selection on ampicillin plates. Genomic DNA of CVC3082 was used in the natural transformation of CVC3022 to generate CVC3101 upon selection on ampicillin and nourseothricin plates. CVC3101 contains the inducible systems to delete *crtS* and to block Chr1 replication by the Tus-*ter* complex.

### Time-lapse fluorescence microscopy

Single colonies grown overnight on LB plates with antibiotics were used to inoculate the supplemented M63 medium and the cultures were grown at 30 °C (for better folding of fluorescent proteins) to log phase (OD_600nm_ 0.3). They were diluted two-fold in the same medium to which 0.2% arabinose or 20 ng/ml aTc was added and the growth was continued 90 min or 45 min, respectively. 5 μl of cell culture was spotted on the center of a glass P35 dish (MatTek corporation, Ashland, MA) and the spot was overlaid with 1% agarose gel pad made with the same supplemented M63 medium. Time-lapse microscopy was performed starting at 90 min after inducer addition, with images taken every 15 min, unless otherwise specified, on a Nikon TiE inverted microscope with Nikon 100x/1.4 Oil Plan Apo Ph3 DM lens, Lumencor sola light engine (Beaverton, OR), and ImageEM EMCCD camera (Hamamatsu, Japan). The fluorescence from GFP was detected using 10% laser output and 300 msec exposure, and from td-Tomato using 30% laser output and 700 msec exposure.

### Time-lapse fluorescence microscopy using microfluidic chamber

Single colonies grown overnight on LB plates were used to inoculate the supplemented M63 medium and the cultures were grown at 30 °C to log phase (OD_600nm_ 0.1). They were diluted 10-fold and loaded onto a CellASIC ONIX B04A Microfluidic Bacteria plate (Millipore, Billerica, MA) primed with the supplemented M63 medium with or without 0.2% arabinose. The experiments were carried out in a CellAsic ONIX2 manifold (Millipore) with 5 μl/h flowrate (2 psi), with aeration at 37 °C and images were taken every 10 min. Subsequent image processing was performed on the Fiji platform as described in the main text.

### Analysis of microscopy data

Microscopy images were processed to subtract background and noise using the Fiji platform [50]. The data from the images (number of foci, cell length etc.) were determined using the MicrobeJ plugin [51]. All statistical analyses were performed using Prism7 (GraphPad Software, CA). Images were captured for each strain, quantified and the data presented as mean (from three biological replicates) ± standard error of mean (SEM). Unpaired two-tailed Student’s *t*-tests were performed on the pairs of triplicate values being compared.

### Analysis of marker frequency

Frequencies of four markers, *ori1*, *ori2*, *ter1* and *ter2*, from exponentially growing cells in LB medium with or without 0.2% arabinose were determined by qPCR using a MJ Research PTC-200 Peltier Thermal Cycler (Bio-rad, Hercules, CA) and a LightCycler 480 SYBR Green I master mix (Roche Life Sciences, IN), as described [12].

### Growth of *E*. *coli* dependent on mini-Chr2 replication

Chemically competent DH5α cells carrying p*rctB* (pTVC14) or p*rctB*L156R (pJJ263, which supplies a DnaK-interaction defective RctBL156R mutant) and either p*crtS* (pBJH188) or the vector (pACYC177) were mixed with 100 ng of p*ori2* (pTVC31). 10 μl of the mixture, after allowing 2 h for expression of drug-resistance in LB medium, was used to inoculate 200 μl of LB supplemented with chloramphenicol (12.5 μg/ml) in 96-well plates. The plates were incubated at 37 °C with shaking in the Synergy HT plate reader (BioTek, USA) and OD at 600nm was monitored.

### Whole genome sequencing

Cells were inoculated in LB medium and grown at 30 °C overnight. Genomic DNA was extracted from one ml of the culture using the DNeasy Tissue Kit (Qiagen, Hilden, Germany). DNA samples were quantified using a Bioanalyzer (Life Technologies, CA) and sequenced by the NCI CCR genomics sequencing core on the Illumina MiSeq platform. 2-5 million reads were obtained per sample, which were trimmed and mapped to the published N16961 reference genome [52] on the CLC genomics workbench (Qiagen).

### Western blotting

Total 4.0 OD units of cells grown in supplemented M63 medium was collected and resuspended in 100 μl of 3x SDS sample buffer, boiled for 10 min and centrifuged for 1 min at 13,000 rpm. Aliquots were loaded onto a precast 8–16% Criterion TGX gel (Bio-Rad, Hercules, CA). Following electrophoresis, the proteins were transferred onto a nitrocellulose membrane and probed with RctB or Tus primary antibodies, as previously described [53].

## Supporting information

S1 Fig Blocking of Chr1 replication results in mis-localization of *ori1* and *ori2* foci. Relative position of the foci with respect to the long axis of the cell (y-axis), plotted against increasing cell length (x-axis). The zero in the y-axis denotes the longitudinal mid-cell position. The localization of *ori1*, usually at the poles, is seen to be distributed throughout the cell body upon Chr1-replication block. *ori2* which is found at mid-cell in smaller cells and quarter-cell in longer cells is also seen to be mis-localized upon Chr1 block. The block to Chr1 results in a block in cell division, as the cell continues to lengthen. Average length of cells in the growth conditions here is 2.8 μm. Under Chr1-replication block, average cell length increases to 4 μm. Σn = 1548 for cells without Chr1 replication block and Σn = 903 for cells with Chr1-replication block, respectively.
(TIF)

S2 Fig Time-lapse microscopy images from movie S1 showing the reversibility of the Chr1 replication-block upon removal of the inducer. The experiments were carried out in a CellAsic ONIX2 manifold using the bacteria plate and were imaged every 10 min. When the inducer (0.2% arabinose) was present (panels 2–8), the *ori1* focus (green spot; black arrow head; panel 1) remained single. During this period, the *ori2* focus (red spot; white arrow head; panel 1) also remained single. Upon removal of the inducer (panels 9–20), foci numbers of both *ori1* and *ori2* increased and cell division resumed (long arrow; panel 17). Scale bars, 2 μm.
(TIF)

S3 Fig Tus concentration reduces upon inducer removal and RctB does not accumulate under Chr1-replication block. (A) Western blot of Tus protein produced in strain CVC3022 upon addition of 0.2% arabinose and upon washing out arabinose. Values below each lane correspond to relative intensity, with respect to that of the loading control, from two replicates. (B) Western blot of RctB protein produced in strain CVC3022 upon addition of 0.2% arabinose and at different times after. Values below each lane correspond to relative intensity, with respect to the amount of RctB at 0’, normalized to the total protein loaded as quantified by SDS-PAGE, from two replicates. Strain used here is same as in Fig 1.
(TIF)

S4 Fig p*crtS* increases Chr2 replication and affects colony size, morphology in *V*. *cholerae*. (A) The presence of p*crtS* but not the vector, causes an increase in the number of *ori2* foci per cell as compared to the vector. Strains used are CVC3171 (vector) and CVC3115 (p*crtS*). Data represents mean ± SEM, averaged from three trials. (B) Presence of p*crtS* also causes reduction colony size (top panel) and change in colony morphology (when in a *hapR*^WT^ unmodified background, bottom panel), giving rise to a rugose appearance. Strains used here are CVC3171 (vector) and CVC3115 (p*crtS*) in the top panel and CVC3210 (vector) and CVC3208 (p*crtS*) in the bottom panel.
(TIF)

S5 Fig p*crtS* does not affect Chr1 replication block by the Tus-*ter* complex. Histograms showing the percentage of cells with the indicated number of *ori1* foci. The figure shows a similar distribution of *ori1* foci (at 150 min after addition of inducer) whether the cells have the empty vector (CVC3145, Σn = 790) or p*crtS* (CVC3028, Σn = 1025). Data represent mean ± SEM of percentages calculated from three biological replicates. Statistical significance was calculated using a Student’s *t*-test. “n.s.” denotes not significant, *p*-value being = 0.4.
(TIF)

S6 Fig The presence of extra copies of *crtS* in Chr1 does not affect the growth of *V*. *cholerae* cells. (A) Schematic of Chr1 showing *crtS* locations in different strains. The locations are at 0.81 Mb (native position) in CVC3058, or at 0.80 and 0.81 Mb in CVC3061, or at 0.81 and 1.84 Mb in CVC3093, or at 0.80, 0.81 and 1.84 Mb in CVC3150 or at 0.80 Mb in CVC3112. The strains are the same as in Fig 3A. (B) Growth curve of strains containing one, two or three copies of *crtS* in the absence of a Chr1 replication-block showing no significant changes in growth rate upon the addition of extra copies of *crtS*. Cells were grown in LB at 37 °C in a 96-well plate. Data represents mean ± SEM from two biological replicates, each performed in duplicate. (C) Average length of cells under log phase of growth (OD_600nm_ 0.2-0.3) shows no significant difference in cell size among the strains tested. When cells with two *ori2* were separately scored, their average length was shorter in strains that had more than one copy of *crtS*, indicating that Chr2 initiates earlier in these strains. Data represents the median with interquartile range where n>1000 cells for each strain.
(TIF)

S7 Fig Summary of time-lapse microscopy experiments in Chr1 replication-blocked cells with either one, two or three *crtS* copies. The Table indicates percentage of Chr2 replication in cells under Chr1-replication block. The number of cells scored are in parentheses. Strains used here are same as in Fig 3C. Cells were imaged every 20 min after addition of the inducer for 30 min to block Chr1 replication and followed from the time a mother cell with two *ori1* foci divided into daughters with one *ori1* focus. Most of these cells were born with one *ori2* focus that either did not duplicate (row 1) or duplicated (row 2) during the course of time-lapse, spanning ~ 150 min. Chr2 replicated in more cells and earlier (row 4) when multiple *crtS* copies were present. Note that a minority (4–9%) of cells were born already with two *ori2* (row 3) when multiple *crtS* copies were present. These *ori2* foci were either inherited from the mother cell or were products of duplication during the initial 20 min interval after the division of the mother cell.
(TIF)

S8 Fig In strains with one, two or three *crtS*, RctB levels do not change upon Chr1-replication block. Western blot of RctB protein produced in strain CVC3022 before addition of 0.2% arabinose and 150 mins after addition in various *V*. *cholerae* strains. Values below each lane correspond to relative intensity, with respect to the amount of RctB at 0’ in CVC3022, normalized to the total protein loaded as quantified by SDS-PAGE, from two replicates. Strains used here are same as in Fig 3C. The increase seen in strain CVC3150 as compared to CVC3022 is probably due to the increased replication of Chr2 observed, resulting in increased gene dosage of the *rctB* gene.
(TIF)

S9 Fig Increasing RctB concentration promotes Chr2 replication in the presence of Chr1 replication. (A) Western blot analysis of RctB protein levels in cells with p*rctB*LOW (CVC3052) and p*rctB*HIGH (CVC3125), shows roughly 1.5 and 9-fold increase, respectively, as compared to cells with no gratuitous source of RctB. (B) Histogram of *ori2* foci number per cell (on x-axis) as the percentage of total cells (on y-axis) under log phase growth (without Chr1 block) in the presence of p*rctB*LOW (CVC3173, Σn = 992), p*rctB*HIGH (CVC3174, Σn = 924) and the empty vector (CVC3171, Σn = 1097). Strains with p*rctB*HIGH show increased number of cells with 2 or more *ori2* foci. Data represent mean ± SEM of percentages calculated from three biological replicates. Statistical significance was calculated using a Student’s *t*-test. “n.s.” denotes *p*-value = 0.0834, hence considered not significant.
(TIF)

S10 Fig *crtS* rescues replication proficiency of p*ori2* with a defective origin and thereby the growth of *E*. *coli* that is dependent on p*ori2* replication. (A) A map of *V*. *cholerae* Chr2 origin region showing insertion of 5 bases in *ori2*. (B) The experiments were performed as in Fig 5 except that a replication-defective *ori2* was used instead of an initiation-defective RctB mutant. The mutant *ori2* has a 5 bp insertion at nt 444 and fails to support stable maintenance of a plasmid dependent on that origin, thereby affect the growth of *E*. *coli* that depends on *ori2* function. Addition of p*crtS* supports *E*. *coli* growth apparently by allowing *ori2* to function. In these experiments, *E*. *coli* containing p*rctB* (pTVC11) and either vector (pACYC177) or p*crtS* (pBJH188), was made chemically competent after growing in the presence of 0.2% arabinose (to induce RctB expression) and transformed with 100 ng of p*ori2* (pTVC210) or p*ori2* that has a 5 bp insertion in *ori2* (pTVC214). Transformants were grown on LB supplemented with ampicillin (to select p*ori2*) and 0.2% arabinose. Growth of *E*. *coli* harboring pTVC214 is supported only in the presence of p*crtS*. The plasmids used here were described previously [11].
(TIF)

S11 Fig A mutant RctB that makes a Δ*crtS* strain viable remains activable by *crtS* for mini-Chr2 replication. (A) Sequence alignment of WT RctB and RctBR423P. The R423P change suppresses growth defect of *ΔcrtS* (CVC2540) strain. The R423 residue is shown in magenta in an α-helix of a winged-helix-turn-helix motif in the dimeric structure of RctB (PDB Id: 5tbf). (B) *crtS* promotes RctBR423P initiator function. The function was assayed by measuring the copy number of a mini-Chr2 (pTVC25) in *E*. *coli*. The cells had either WT RctB or RctBR423P and p*crtS* or the corresponding empty vector. A representative gel from three biological replicates (left) and their quantification (right) are shown. The mini-Chr2 band intensities were first normalized to p*crtS* or vector bands and the resulting values were further normalized by setting the mini-Chr2 copy number in the presence of WT RctB to 1 (dashed line). p*crtS* increased mini-Chr2 copy number in the presence of both the initiators, indicating that the mutant is still responsive to *crtS*.
(TIF)

S12 Fig Time-lapse microscopy images from movie S2 showing the failure of Chr2 replication upon excision of *crtS*. CVC3082 (same as in Fig 5) was induced with 20 ng/ml aTc for 45 min prior to the start of the time-lapse. Images were taken every 10 min. Upon induction of the integrase by aTc, cells continued to divide but duplication of *ori2* foci ceased, giving rise to daughter cells lacking any *ori2* focus (panels 5–19). Scale bar, 2 μm.
(TIF)

S1 Movie Time-lapse movie showing reversibility of Chr1 replication-block upon removal of the inducer. (AVI)

S2 Movie Failure of Chr2 replication upon excision of *crtS*. (AVI)

S1 Table Bacterial strains used in this study. (DOCX)

S2 Table Plasmids used in this study. (DOCX)

S3 Table Primers used in this study. (DOCX)
