# Supplementary figures and images for: Chromosome 1 licenses chromosome 2 replication in Vibrio cholerae by doubling the crtS gene dosage
Source: PLoS Genet. 2018 May 24;14(5):e1007426. doi: 10.1371/journal.pgen.1007426 (PMC5991422; doi:10.1371/journal.pgen.1007426)

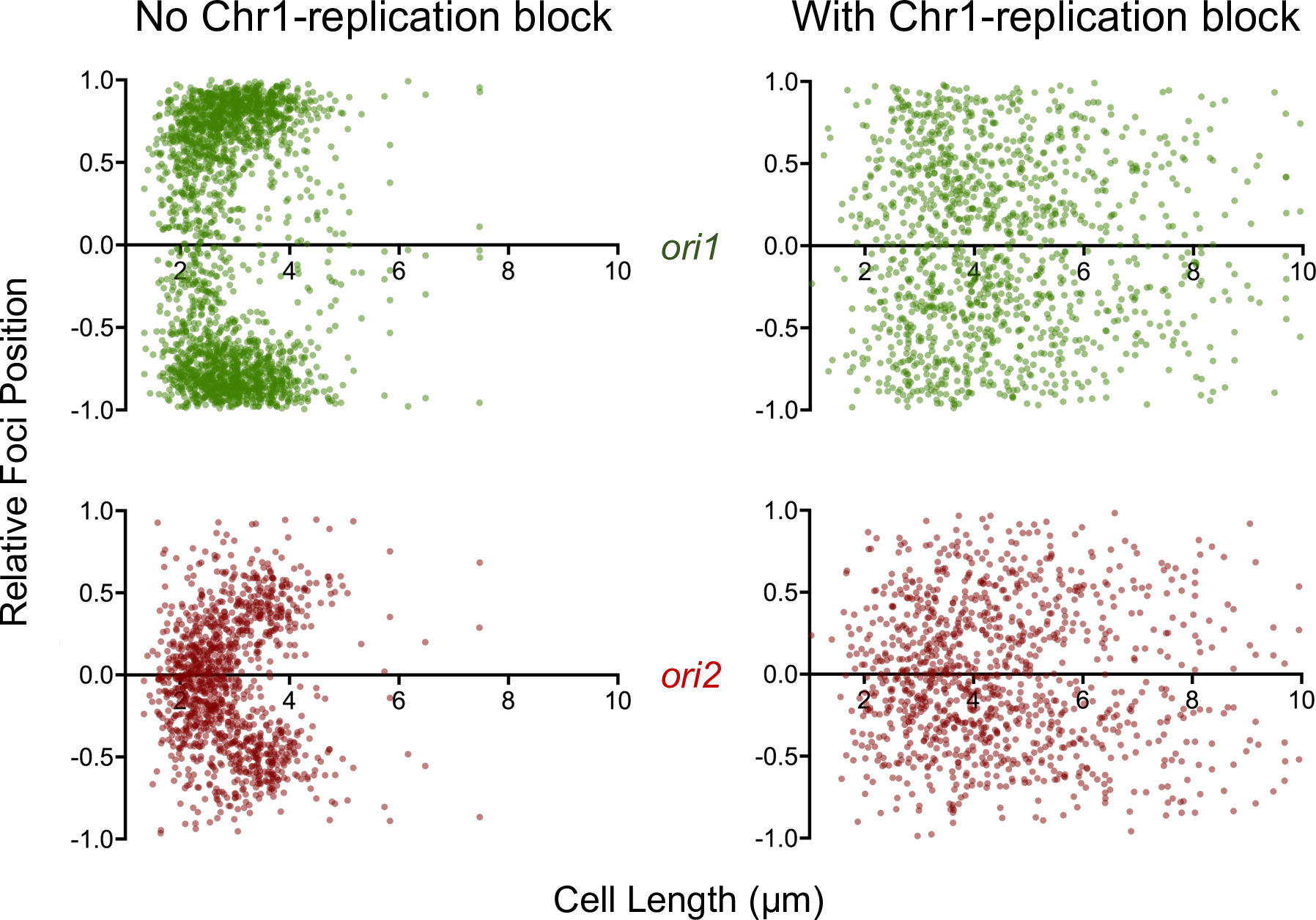

Supplement: S1 Fig — Relative position of the foci with respect to the long axis of the cell (y-axis), plotted against increasing cell length (x-axis). The zero in the y-axis denotes the longitudinal mid-cell position. The localization of ori1, usually at the poles, is seen to be distributed throughout the cell body upon Chr1-replication block. ori2 which is found at mid-cell in smaller cells and quarter-cell in longer cells is also seen to be mis-localized upon Chr1 block. The block to Chr1 results in a block in cell division, as the cell continues to lengthen. Average length of cells in the growth conditions here is 2.8 μm. Under Chr1-replication block, average cell length increases to 4 μm. Σn = 1548 for cells without Chr1 replication block and Σn = 903 for cells with Chr1-replication block, respectively. (TIF) [file pgen.1007426.s001.tif]

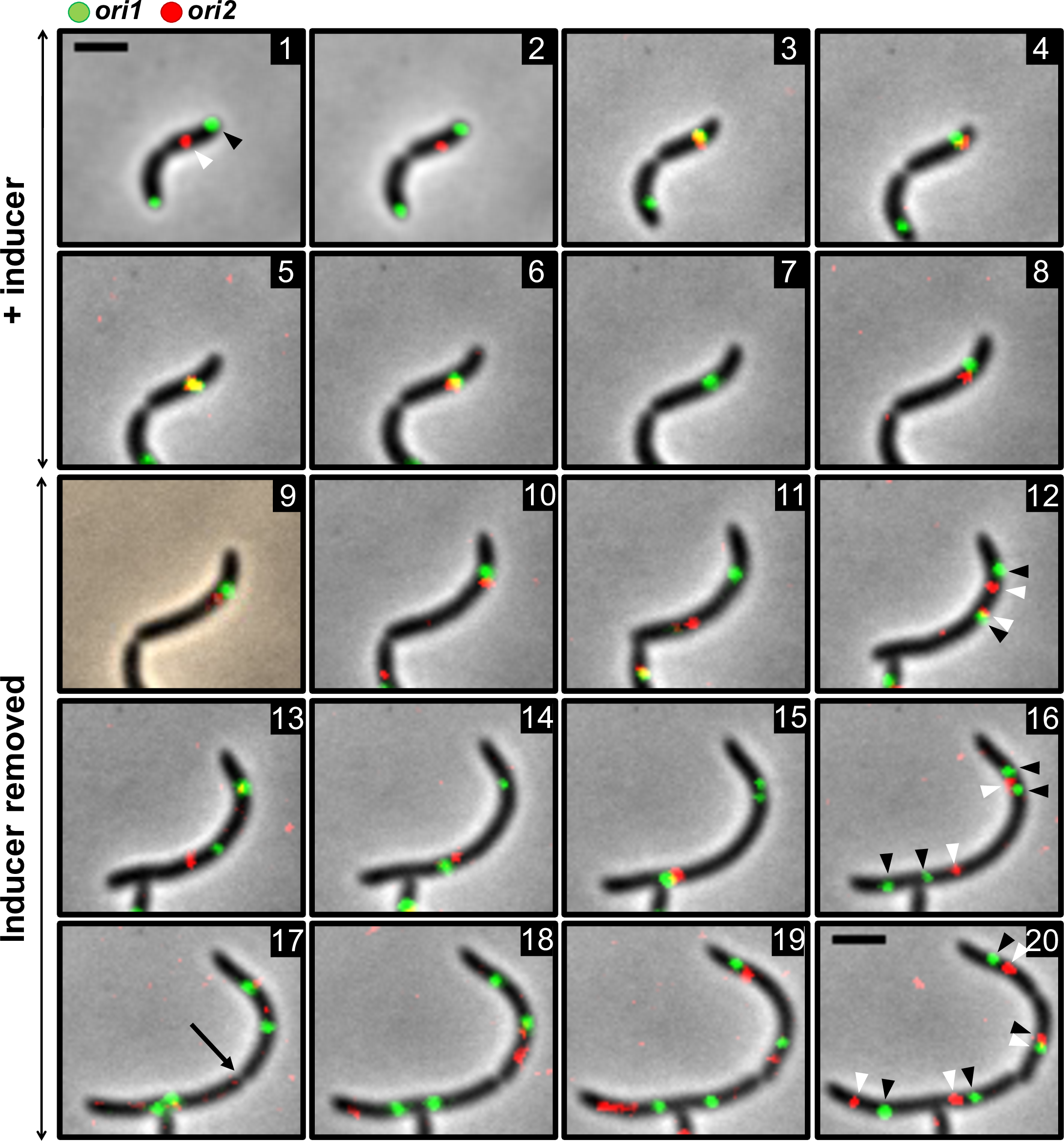

Supplement: S2 Fig — The experiments were carried out in a CellAsic ONIX2 manifold using the bacteria plate and were imaged every 10 min. When the inducer (0.2% arabinose) was present (panels 2–8), the ori1 focus (green spot; black arrow head; panel 1) remained single. During this period, the ori2 focus (red spot; white arrow head; panel 1) also remained single. Upon removal of the inducer (panels 9–20), foci numbers of both ori1 and ori2 increased and cell division resumed (long arrow; panel 17). Scale bars, 2 μm. (TIF) [file pgen.1007426.s002.tif]

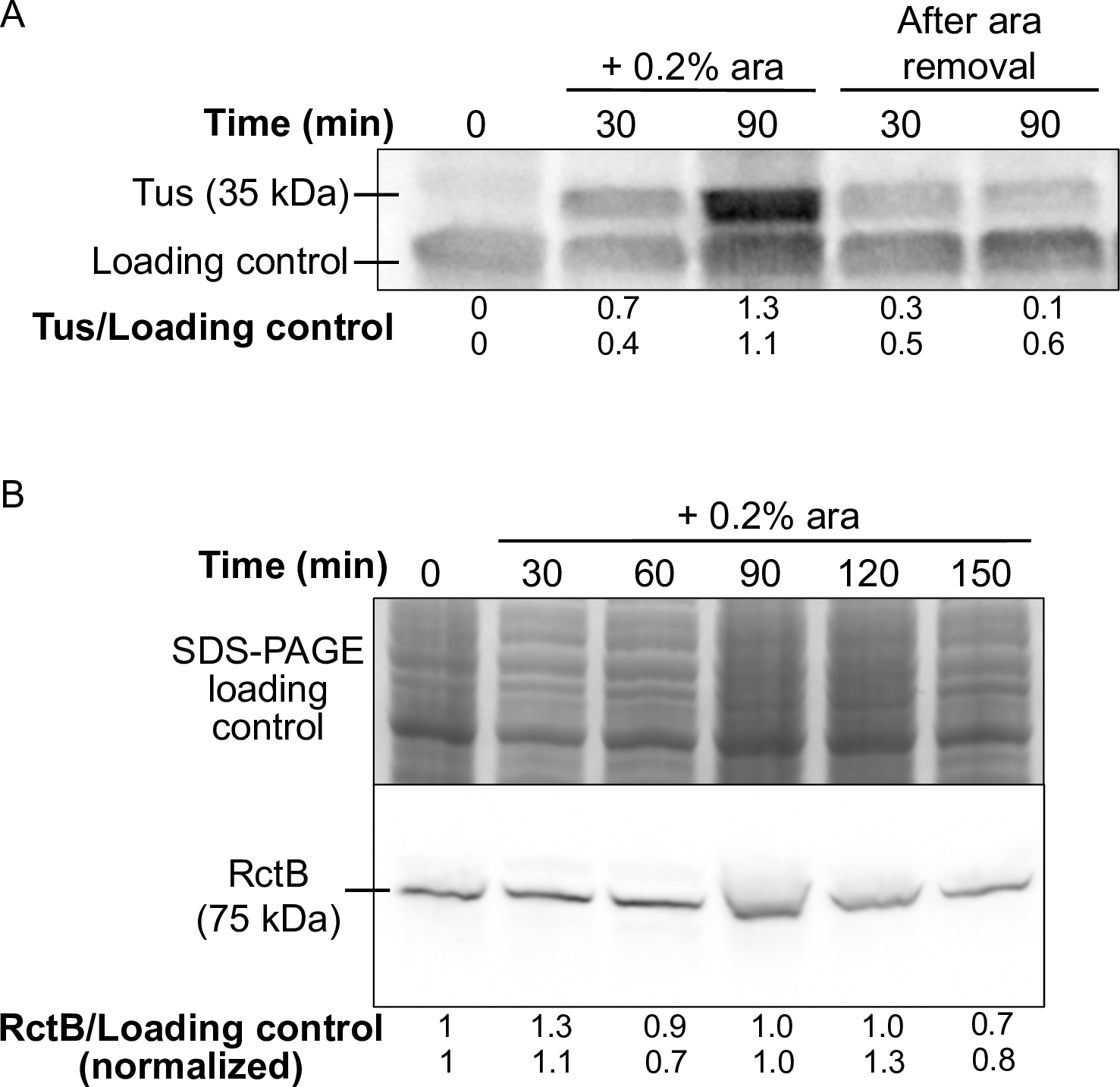

Supplement: S3 Fig — (A) Western blot of Tus protein produced in strain CVC3022 upon addition of 0.2% arabinose and upon washing out arabinose. Values below each lane correspond to relative intensity, with respect to that of the loading control, from two replicates. (B) Western blot of RctB protein produced in strain CVC3022 upon addition of 0.2% arabinose and at different times after. Values below each lane correspond to relative intensity, with respect to the amount of RctB at 0’, normalized to the total protein loaded as quantified by SDS-PAGE, from two replicates. Strain used here is same as in Fig 1. (TIF) [file pgen.1007426.s003.tif]

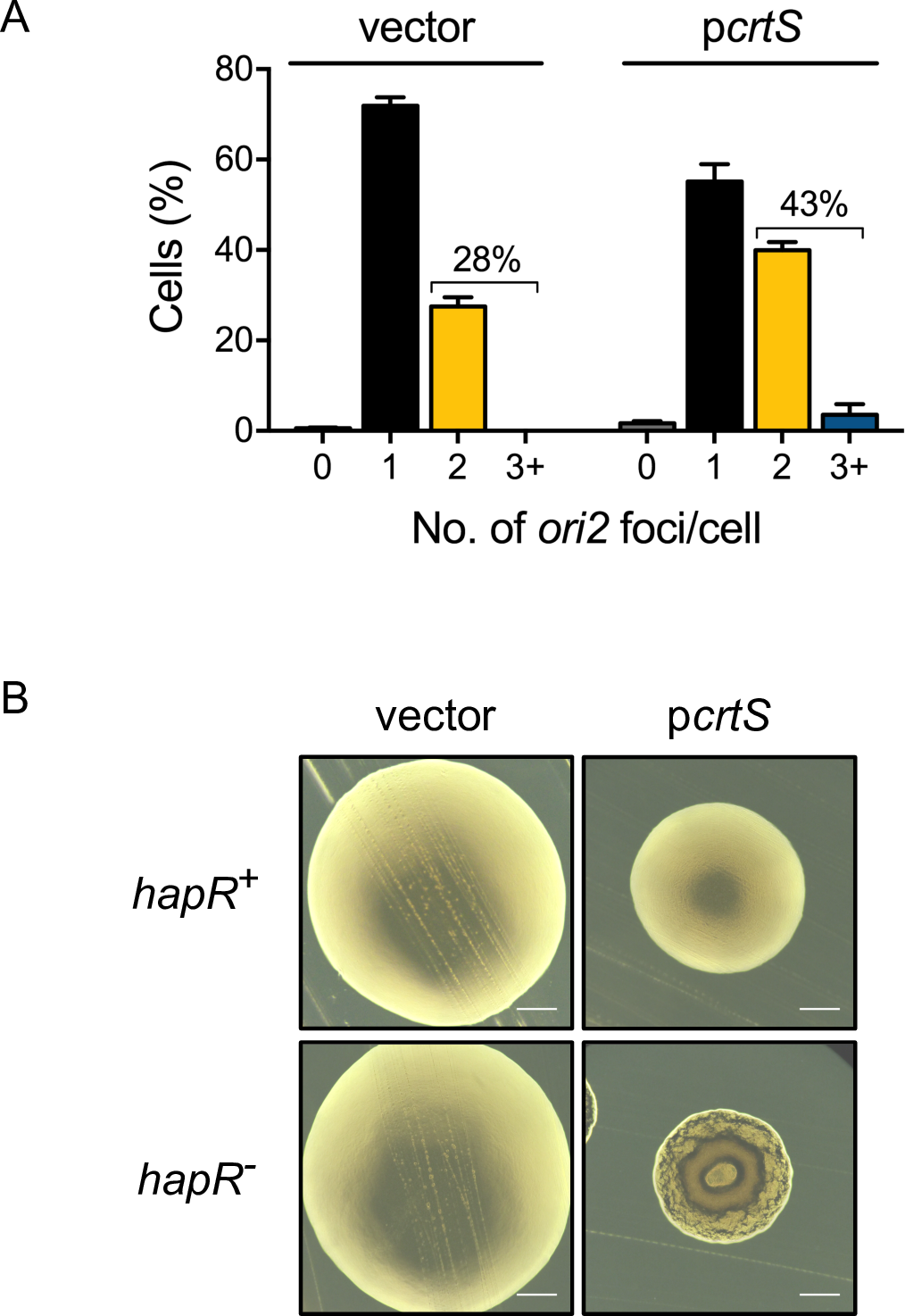

Supplement: S4 Fig — (A) The presence of pcrtS but not the vector, causes an increase in the number of ori2 foci per cell as compared to the vector. Strains used are CVC3171 (vector) and CVC3115 (pcrtS). Data represents mean ± SEM, averaged from three trials. (B) Presence of pcrtS also causes reduction colony size (top panel) and change in colony morphology (when in a hapRWT unmodified background, bottom panel), giving rise to a rugose appearance. Strains used here are CVC3171 (vector) and CVC3115 (pcrtS) in the top panel and CVC3210 (vector) and CVC3208 (pcrtS) in the bottom panel. (TIF) [file pgen.1007426.s004.tif]

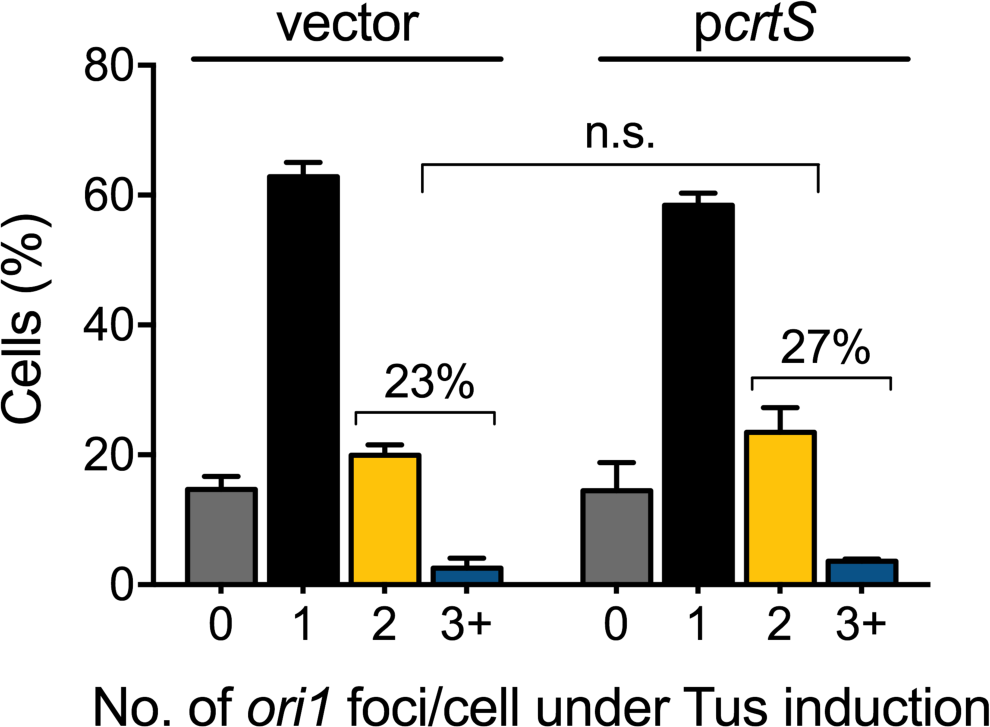

Supplement: S5 Fig — Histograms showing the percentage of cells with the indicated number of ori1 foci. The figure shows a similar distribution of ori1 foci (at 150 min after addition of inducer) whether the cells have the empty vector (CVC3145, Σn = 790) or pcrtS (CVC3028, Σn = 1025). Data represent mean ± SEM of percentages calculated from three biological replicates. Statistical significance was calculated using a Student’s t-test. “n.s.” denotes not significant, p-value being = 0.4. (TIF) [file pgen.1007426.s005.tif]

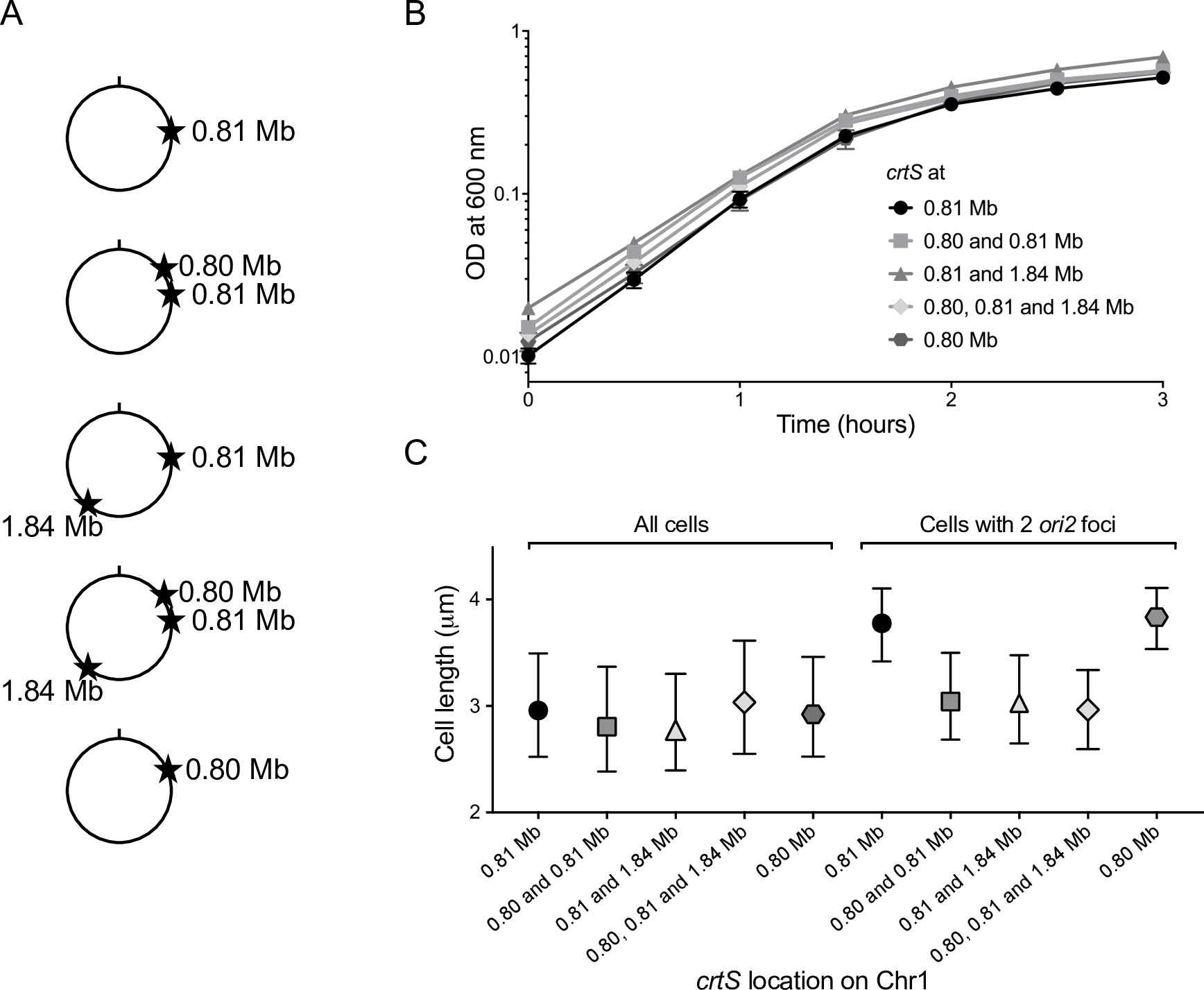

Supplement: S6 Fig — (A) Schematic of Chr1 showing crtS locations in different strains. The locations are at 0.81 Mb (native position) in CVC3058, or at 0.80 and 0.81 Mb in CVC3061, or at 0.81 and 1.84 Mb in CVC3093, or at 0.80, 0.81 and 1.84 Mb in CVC3150 or at 0.80 Mb in CVC3112. The strains are the same as in Fig 3A. (B) Growth curve of strains containing one, two or three copies of crtS in the absence of a Chr1 replication-block showing no significant changes in growth rate upon the addition of extra copies of crtS. Cells were grown in LB at 37 °C in a 96-well plate. Data represents mean ± SEM from two biological replicates, each performed in duplicate. (C) Average length of cells under log phase of growth (OD600nm 0.2-0.3) shows no significant difference in cell size among the strains tested. When cells with two ori2 were separately scored, their average length was shorter in strains that had more than one copy of crtS, indicating that Chr2 initiates earlier in these strains. Data represents the median with interquartile range where n>1000 cells for each strain. (TIF) [file pgen.1007426.s006.tif]

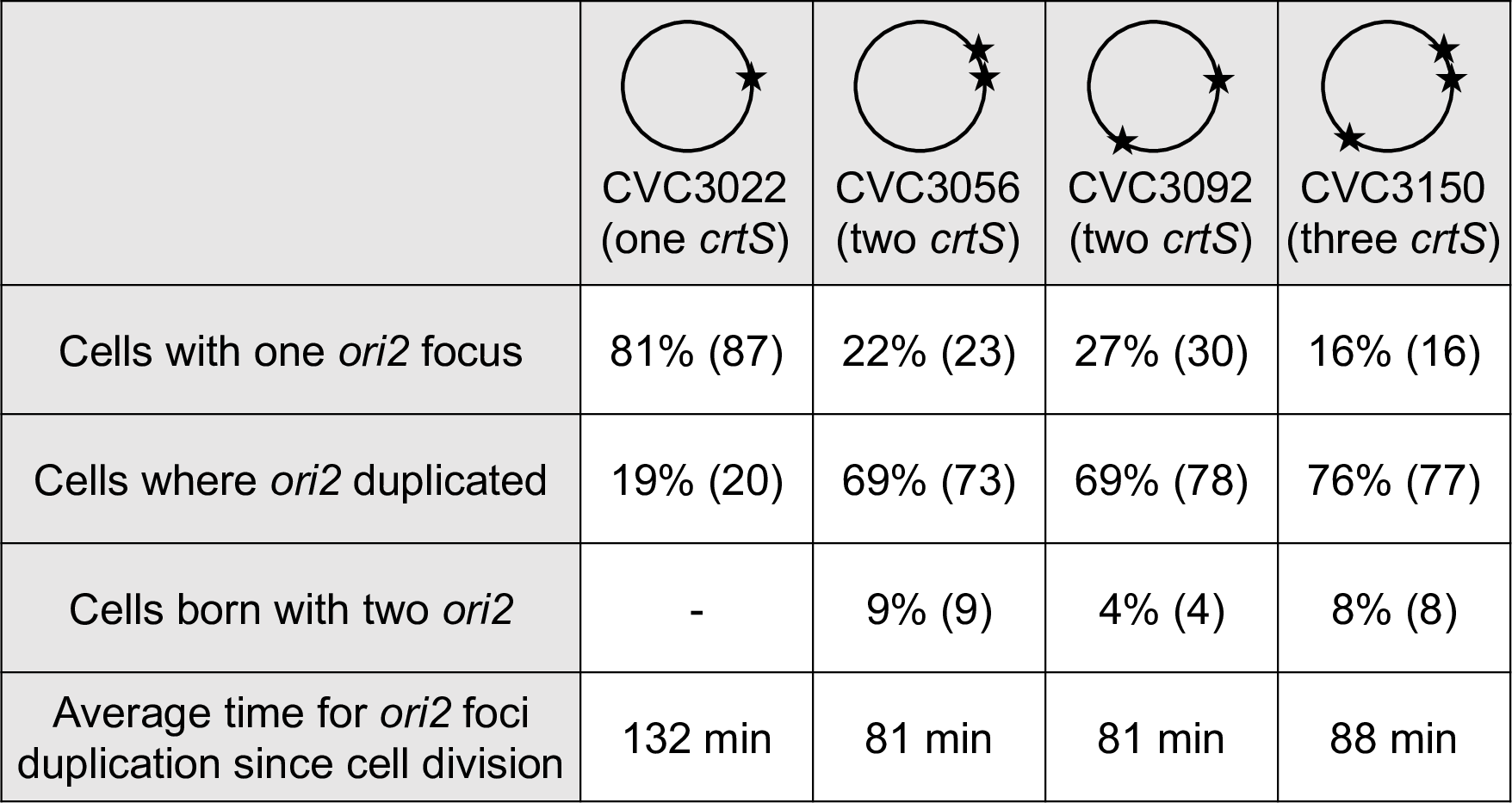

Supplement: S7 Fig — The Table indicates percentage of Chr2 replication in cells under Chr1-replication block. The number of cells scored are in parentheses. Strains used here are same as in Fig 3C. Cells were imaged every 20 min after addition of the inducer for 30 min to block Chr1 replication and followed from the time a mother cell with two ori1 foci divided into daughters with one ori1 focus. Most of these cells were born with one ori2 focus that either did not duplicate (row 1) or duplicated (row 2) during the course of time-lapse, spanning ~ 150 min. Chr2 replicated in more cells and earlier (row 4) when multiple crtS copies were present. Note that a minority (4–9%) of cells were born already with two ori2 (row 3) when multiple crtS copies were present. These ori2 foci were either inherited from the mother cell or were products of duplication during the initial 20 min interval after the division of the mother cell. (TIF) [file pgen.1007426.s007.tif]

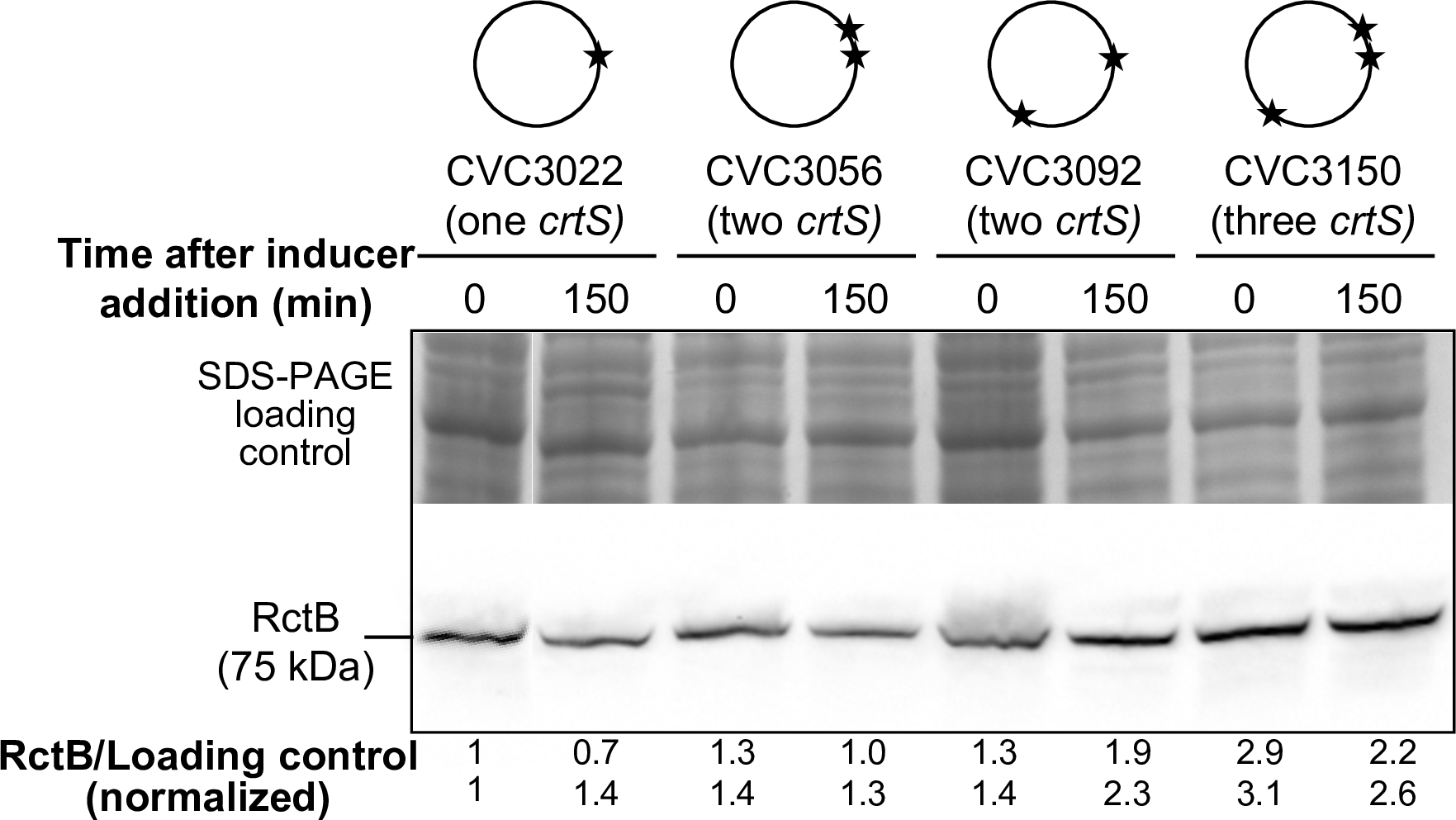

Supplement: S8 Fig — Western blot of RctB protein produced in strain CVC3022 before addition of 0.2% arabinose and 150 mins after addition in various V. cholerae strains. Values below each lane correspond to relative intensity, with respect to the amount of RctB at 0’ in CVC3022, normalized to the total protein loaded as quantified by SDS-PAGE, from two replicates. Strains used here are same as in Fig 3C. The increase seen in strain CVC3150 as compared to CVC3022 is probably due to the increased replication of Chr2 observed, resulting in increased gene dosage of the rctB gene. (TIF) [file pgen.1007426.s008.tif]

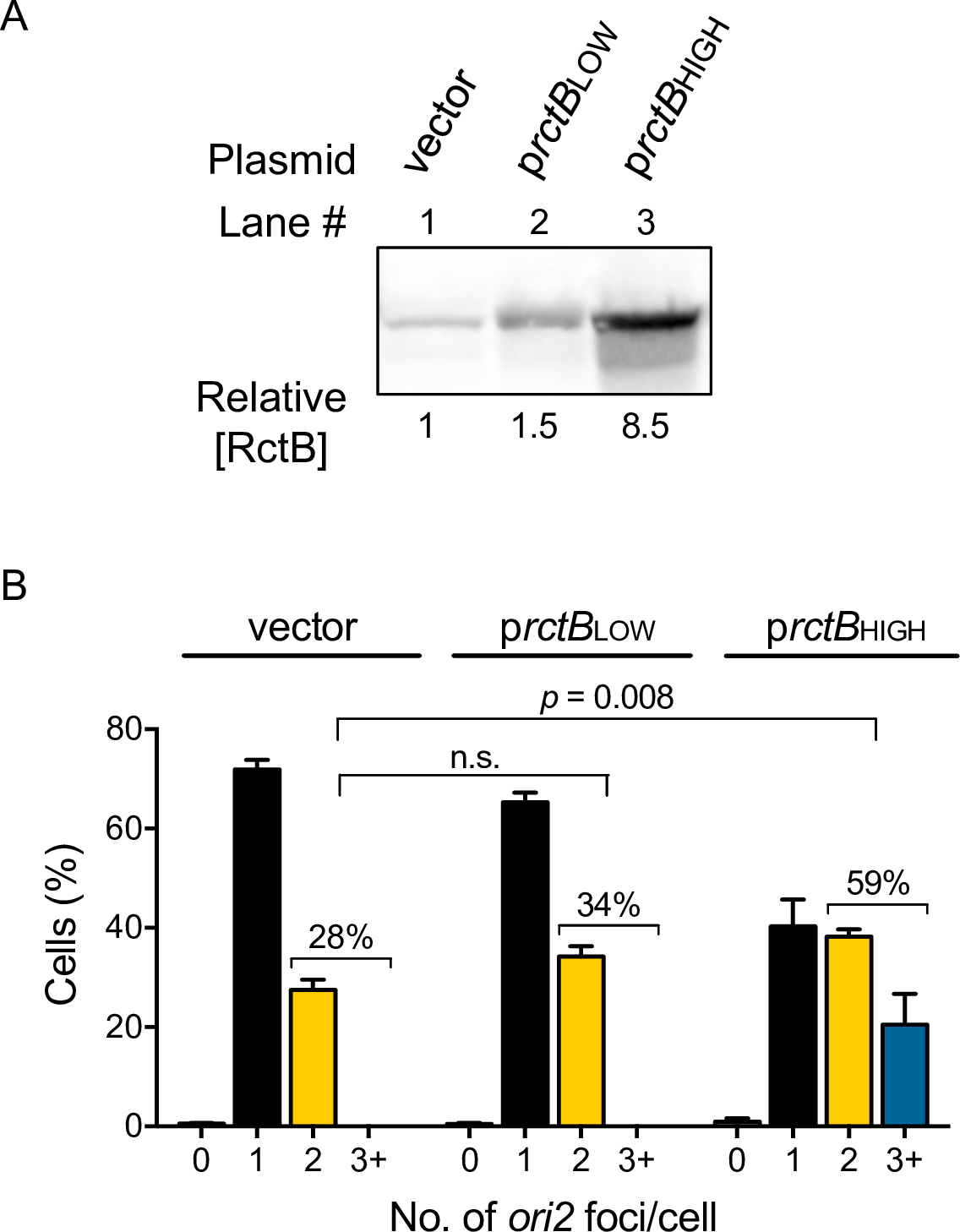

Supplement: S9 Fig — (A) Western blot analysis of RctB protein levels in cells with prctBLOW (CVC3052) and prctBHIGH (CVC3125), shows roughly 1.5 and 9-fold increase, respectively, as compared to cells with no gratuitous source of RctB. (B) Histogram of ori2 foci number per cell (on x-axis) as the percentage of total cells (on y-axis) under log phase growth (without Chr1 block) in the presence of prctBLOW (CVC3173, Σn = 992), prctBHIGH (CVC3174, Σn = 924) and the empty vector (CVC3171, Σn = 1097). Strains with prctBHIGH show increased number of cells with 2 or more ori2 foci. Data represent mean ± SEM of percentages calculated from three biological replicates. Statistical significance was calculated using a Student’s t-test. “n.s.” denotes p-value = 0.0834, hence considered not significant. (TIF) [file pgen.1007426.s009.tif]

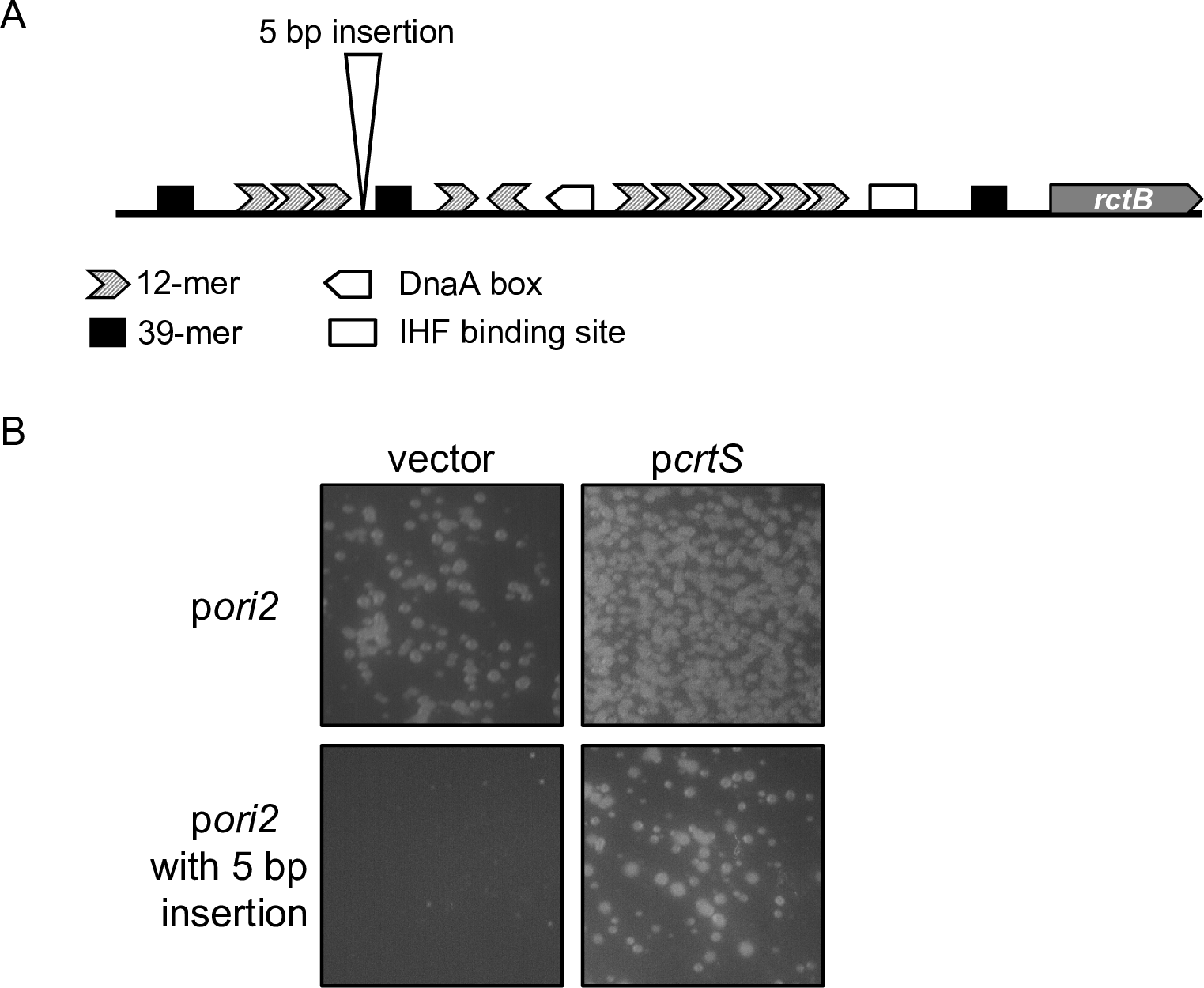

Supplement: S10 Fig — (A) A map of V. cholerae Chr2 origin region showing insertion of 5 bases in ori2. (B) The experiments were performed as in Fig 5 except that a replication-defective ori2 was used instead of an initiation-defective RctB mutant. The mutant ori2 has a 5 bp insertion at nt 444 and fails to support stable maintenance of a plasmid dependent on that origin, thereby affect the growth of E. coli that depends on ori2 function. Addition of pcrtS supports E. coli growth apparently by allowing ori2 to function. In these experiments, E. coli containing prctB (pTVC11) and either vector (pACYC177) or pcrtS (pBJH188), was made chemically competent after growing in the presence of 0.2% arabinose (to induce RctB expression) and transformed with 100 ng of pori2 (pTVC210) or pori2 that has a 5 bp insertion in ori2 (pTVC214). Transformants were grown on LB supplemented with ampicillin (to select pori2) and 0.2% arabinose. Growth of E. coli harboring pTVC214 is supported only in the presence of pcrtS. The plasmids used here were described previously [11]. (TIF) [file pgen.1007426.s010.tif]

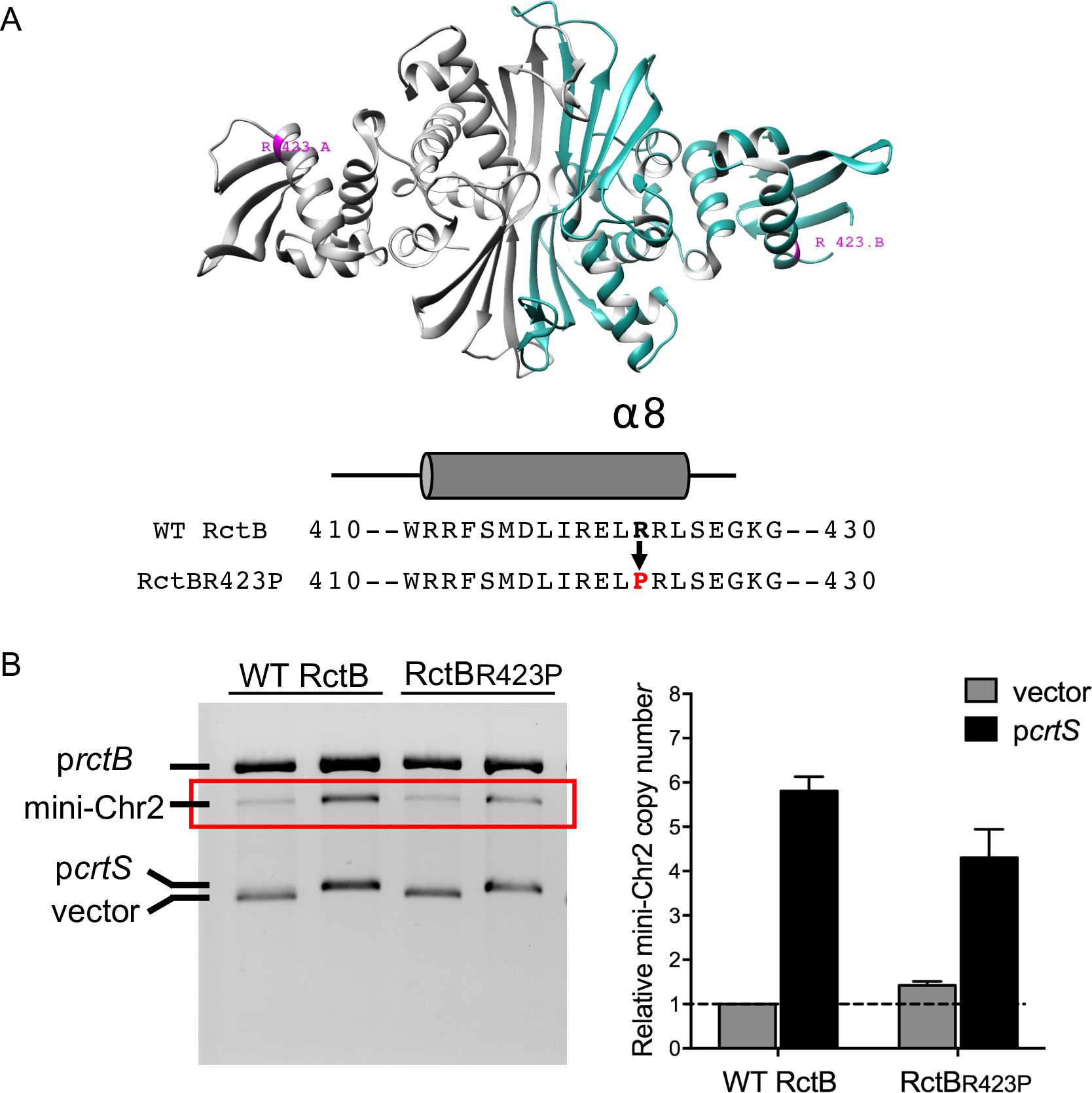

Supplement: S11 Fig — (A) Sequence alignment of WT RctB and RctBR423P. The R423P change suppresses growth defect of ΔcrtS (CVC2540) strain. The R423 residue is shown in magenta in an α-helix of a winged-helix-turn-helix motif in the dimeric structure of RctB (PDB Id: 5tbf). (B) crtS promotes RctBR423P initiator function. The function was assayed by measuring the copy number of a mini-Chr2 (pTVC25) in E. coli. The cells had either WT RctB or RctBR423P and pcrtS or the corresponding empty vector. A representative gel from three biological replicates (left) and their quantification (right) are shown. The mini-Chr2 band intensities were first normalized to pcrtS or vector bands and the resulting values were further normalized by setting the mini-Chr2 copy number in the presence of WT RctB to 1 (dashed line). pcrtS increased mini-Chr2 copy number in the presence of both the initiators, indicating that the mutant is still responsive to crtS. (TIF) [file pgen.1007426.s011.tif]

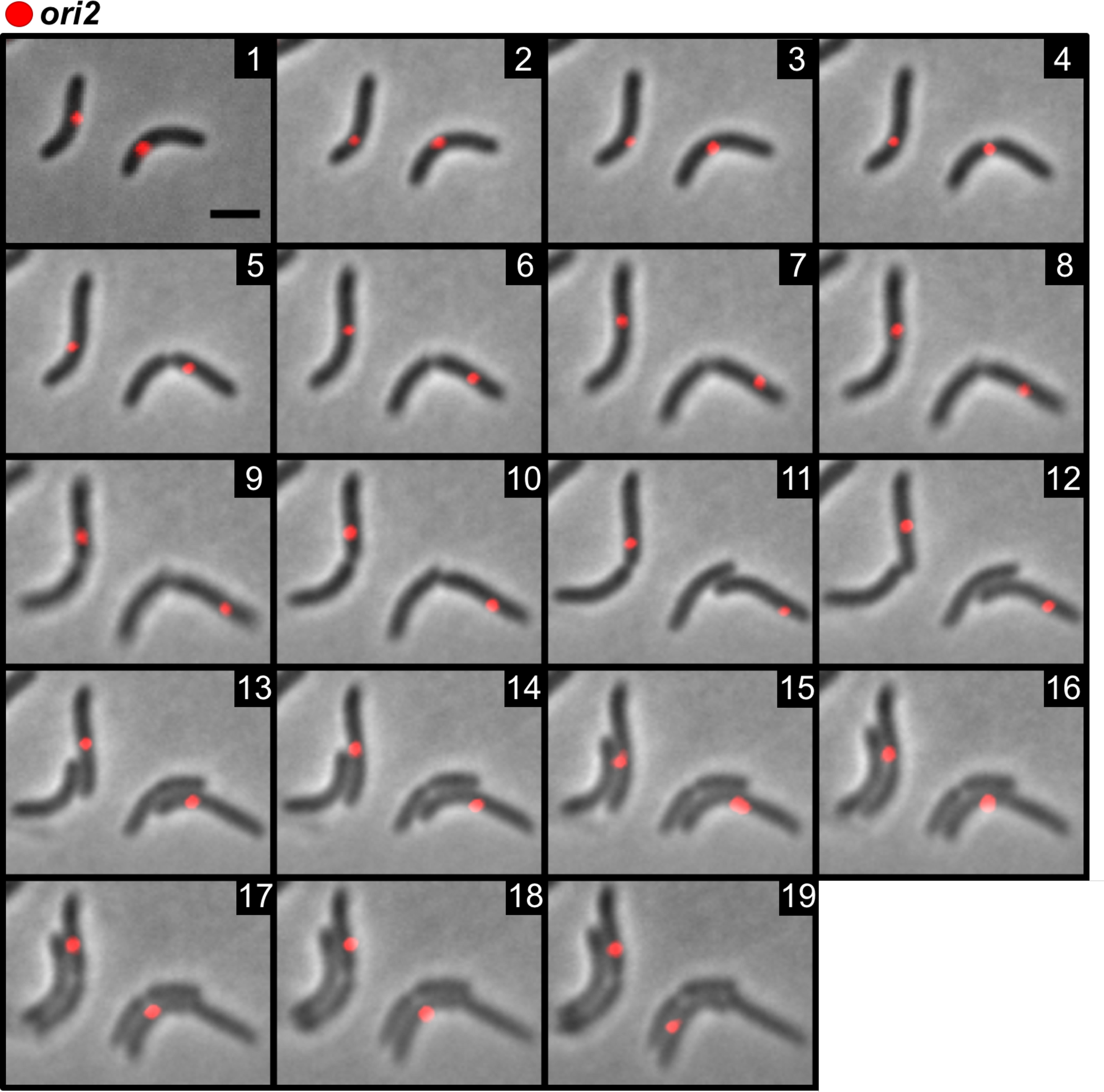

Supplement: S12 Fig — CVC3082 (same as in Fig 5) was induced with 20 ng/ml aTc for 45 min prior to the start of the time-lapse. Images were taken every 10 min. Upon induction of the integrase by aTc, cells continued to divide but duplication of ori2 foci ceased, giving rise to daughter cells lacking any ori2 focus (panels 5–19). Scale bar, 2 μm. (TIF) [file pgen.1007426.s012.tif]
