# Supplementary material for: Chromosome 1 licenses chromosome 2 replication in Vibrio cholerae by doubling the crtS gene dosage
Source: PLoS Genet. 2018 May 24;14(5):e1007426. doi: 10.1371/journal.pgen.1007426 (PMC5991422; doi:10.1371/journal.pgen.1007426)
Supplement: S1 Table — (DOCX) [file pgen.1007426.s015.docx]

**S1 Table Bacterial strains used in this study**

| **Strains** | **Relevant Characteristics** | **Source/Figure** |
| --- | --- | --- |
| CVC209 | *V. cholerae* El Tor N16961; Str^R^ | M. Waldor |
| CVC1121 | N16961 *hapR^+^ ∆dns*; Str^R^, Gm^R^ | M. Blokesch |
| CVC1157 | CVC1121 *terB-ori1-zeo-terB*; Gm^R^, Zeo^R^ | R. Kadoya |
| CVC2540 | CVC1121 ∆*crtS* FRT‑*zeo‑*FRT; Zeo^R^ | [1] |
| CVC3001 | CVC1121 *lacZ*::(*araC pbad-tus* *lacI*^q^ *ptac*-(*tdTomato-*pMT*parB gfp-*P1*parB t1t2*), *cat*); Gm^R^, Cm^R^ | This study |
| CVC3009 | CVC3001 P1*parS* Kn (at +135 kb on Chr1) pMT*parS* Sp (at +40 kb on Chr2); Gm^R^, Cm^R^, Kn^R^, Sp^R^ | This study |
| CVC3013 | CVC1121 *lacZ*::*kanR2*; Kn^R^ | This study |
| CVC3019 | CVC3009 *terCB* *ori1* z*eo* *terBC*; Gm^R^, Cm^R^, Kn^R^, Sp^R^, Zeo^R^ | Fig 1C |
| CVC3022 | CVC3019 ∆Kn cassette; Gm^R^, Cm^R^, Sp^R^, Zeo^R^ | Figs 1B,D,E, 3C,  S1,S2,S3,S7,S8 Figs |
| CVC3028 | CVC3022/p*crtS*; Gm^R^, Cm^R^, Kn^R^, Sp^R^, Zeo^R^ | Fig 2A,B, S5 Fig |
| CVC3052 | CVC3022/p*rctB*LOW (pTVC13); Gm^R^, Cm^R^, Sp^R^, Zeo^R^, Kn^R^ | Fig 4A,B, S9A Fig |
| CVC3056 | CVC3019 *crtS-nat* (at 0.80 Mb); Gm^R^, Cm^R^, Kn^R^, Sp^R^, Zeo^R^, Nat^R^ | Fig 3B,C, S7,S8 Figs |
| CVC3058 | CVC1121 P1*parS* (at +40 kb on Chr2) *lacZ*::(*araC pbad-tus* *lacI*^q^ *ptac*-(*tdTomato-*pMT*parB gfp-*P1*parB t1t2*), *cat*); Gm^R^, Cm^R^ | Fig 3A, S6B,C Fig |
| CVC3061 | CVC3058 *crtS-nat* (at 0.80 Mb); Gm^R^, Cm^R^, Nat^R^ | Fig 3A, S6B,C Fig |
| CVC3068 | CVC3058 *attP-crtS-nat-attB*; Gm^R^, Cm^R^, Nat^R^ | This study |
| CVC3069 | CVC3022 *attP-crtS-nat-attB*; Gm^R^, Cm^R^, Sp^R^, Zeo^R^, Nat^R^ | This study |
| CVC3082 | CVC3068 *bla tetR ptet-*phiC31*int* (at 0.80 Mb, 10 kb upstream of *crtS*; Gm^R^, Cm^R^, Nat^R^, Ap^R^ | Fig 6B,C, S12 Fig |
| CVC3092 | CVC3022 *crtS nat* (at 1.7 Mb); Gm^R^, Cm^R^, Sp^R^, Zeo^R^, Nat^R^ | Fig 3B,C, S7, S8 Figs |
| CVC3093 | CVC3058 *crtS nat* (at 1.7 Mb); Gm^R^, Cm^R^, Nat^R^ | Fig 3A, S6B,C Fig |
| CVC3101 | CVC3069 *bla tetR ptet-*phiC31*int* (at 0.80 Mb,10 kb upstream of *crtS*); Gm^R^, Cm^R^, Sp^R^, Zeo^R^, Nat^R^, Ap^R^ | This study |
| CVC3112 | CVC3061 ∆*crtS*; Gm^R^, Cm^R^, Nat^R^, Zeo^R^ | Fig 3A |
| CVC3115 | CVC3058/pBJH188; Gm^R^, Cm^R^, Nat^R^, Ap^R^, Kn^R^ | S4A,B Fig |
| CVC3125 | CVC3022/p*rctB*HIGH (pRR24); Gm^R^, Cm^R^, Sp^R^, Zeo^R^, Kn^R^ | Fig 4A,B, S9A Fig |
| CVC3137 | CVC3082/pBJH188; Gm^R^, Cm^R^, Nat^R^, Ap^R^, Kn^R^ | Fig 6B,C |
| CVC3150 | CVC3092 *crtS*-Kn (at 0.80 Mbp); Gm^R^, Cm^R^, Sp^R^, Zeo^R^, Nat^R^, Kn^R^ | Fig 3B,C, S7, S8 Figs |
| CVC3151 | CVC3093 *crtS*-Kn (at 0.80 Mbp); Gm^R^, Cm^R^, Nat^R^, Kn^R^ | Fig 3A, S6B,C Fig |
| CVC3164 | CVC3022/pACYC177; Gm^R^, Cm^R^, Sp^R^, Zeo^R^, Kn^R^ | Fig 2B, Fig 4B,  S5, S9A Figs |
| CVC3167 | CVC3101/p*rctB*LOW (pTVC13); Gm^R^, Cm^R^, Sp^R^, Zeo^R^, Nat^R^, Ap^R^, Kn^R^ | Fig 7 |
| CVC3168 | CVC3101/p*rctB*HIGH (pRR24); Gm^R^, Cm^R^, Sp^R^, Zeo^R^, Nat^R^, Ap^R^, Kn^R^ | Fig 7 |
| CVC3169 | CVC3101/pACYC177; Gm^R^, Cm^R^, Sp^R^, Zeo^R^, Nat^R^, Ap^R^, Kn^R^ | Fig 7 |
| CVC3171 | CVC3058/pACYC177; Gm^R^, Cm^R^, Kn^R^ | S4B, S9B Figs |
| CVC3173 | CVC3058/p*rctB*LOW (pTVC13); Gm^R^, Cm^R^, Kn^R^ | S9B Fig |
| CVC3174 | CVC3058/p*rctB*HIGH (pRR24); Gm^R^, Cm^R^, Kn^R^ | S9B Fig |
| CVC3208 | CVC209/pBJH188; Kn^R^ | S4B Fig |
| CVC3210 | CVC209/pACYC177; Kn^R^ | S4B Fig |

1. Baek JH, Chattoraj DK. Chromosome I Controls Chromosome II Replication in *Vibrio cholerae*. PLoS Genet. 2014;10(2):e1004184. doi: 10.1371/journal.pgen.1004184. PubMed PMID: 24586205; PubMed Central PMCID: PMC3937223.
