# Supplementary material for: Chromosome 1 licenses chromosome 2 replication in Vibrio cholerae by doubling the crtS gene dosage
Source: PLoS Genet. 2018 May 24;14(5):e1007426. doi: 10.1371/journal.pgen.1007426 (PMC5991422; doi:10.1371/journal.pgen.1007426)
Supplement: S2 Table — (DOCX) [file pgen.1007426.s016.docx]

**S2 Table Plasmids used in this study**

| **Name** | | **Description** | **Source/Figure** |
| --- | --- | --- | --- |
| pACYC177 |  | Vector; p15A*ori*; Ap^R^, Kn^R^ | NEB, Ipswich, MA |
| pAM101 |  | Source of *nat* gene; pBR*ori*; Ap^R^, Nat^R^ | [1] |
| pASK-IBA32 |  | Vector, source of *ptet*; f1*ori*; Ap^R^ | IBA life sciences, Germany |
| pBJH188 = p*crtS* | | pACYC177∆*bla crtS* (817947-818099); p15A*ori*; Kn^R^ | [2] ; Fig 2, S4, S5, S10, S11 Figs |
| pBJH245 |  | 1 kb homology regions upstream and downstream of *crtS* with a Zeo cassette in place of *crtS*; pBR*ori;* Zeo^R^, Ap^R^ | [2] |
| pGEM-T-easy |  | Vector; pBR*ori*; Ap^R^ | Promega, WI |
| pInt |  | Source of phiC31 *int* gene; p15A*ori*; Kn^R^ | Addgene Plasmid #18941 |
| pJJ114=mini-Chr2 | | Contains *ori2* (775–1133) (same as pTVC31 except for the drug marker); R6K*oriγ*; Cm^R^ | [3] ; S11 Fig |
| pJJ263=p*rctB*L156R | | Source of DnaK-sensitive RctB (same as pTVC14 except for *rctB* mutation causing L156R change); pBR*ori*; Ap^R^ | [3] ; Fig 5 |
| pPS64 |  | pDS132 with 1 kb homology regions upstream and downstream at 1.84 Mb; R6K*oriγ*; Ap^R^ | Preeti Srivastava |
| pRR06 |  | Contains *lacZ*::(*araC pbad-tus* *lacI*^q^ *ptac*-(*tdTomato-*pMT*parB gfp-*P1*parB t1t2*), *cat*) in pGEM-T-easy; pBR*ori;* Cm^R^, Ap^R^ | This study |
| pRR08 |  | *terCB‑ori1‑zeo‑terBC* with 1 kb *ori1* flanking regions cloned in pGEM-T-easy; pBR*ori;* Zeo^R^, Ap^R^ | This study |
| pRR13 |  | P1*parS* Kn with 1 kb flanks in pGEM-T-easy for insertion at 0.80 Mb (10 kb upstream of *crtS*); pBR*ori;* Kn^R^, Ap^R^ | This study |
| pRR16 |  | *crtS-nat*  with 1kb flanks in pRR13 for insertion at 10 kb upstream of c*rtS*; pBR*ori;* Nat^R^, Ap^R^ | This study |
| pRR17 |  | *attP-crtS-nat-attB* in place of Zeo cassette in pBJH245; pBR*ori;* Nat^R^, Ap^R^ | This study |
| pRR20 |  | *crtS-nat* with 1 kb flanks in pPS64 for inserting *crtS* at 1.84 Mb; pBR*ori;* Nat^R^, Ap^R^ | This study |
| pRR21 |  | (*bla tetR* *ptet-*phiC31*int*) with 1 kb flanks in pRR13 for insertion at 0.80 Mb on Chr1; pBR*ori;* Nat^R^, Ap^R^ | This study |
| pRR24= p*rctB*HIGH | | RctB source where *rctB* is transcriptionally fused to *kanR* gene in pACYC177; p15A*ori*; Kn^R^ | Figs. 4, 7, S9 Fig |
| pRR25 |  | *nat* replaced with Kn in pRR16; pBR*ori;* Kn^R^, Ap^R^ | This study |
| pRR26=p*rctB*R423P | | Same as pTVC14 except for *rctB* (R423P); pBR*ori*; pSC101*ori*; Ap^R^ | S11 Fig |
| pTF6 | | *attP-*p15A*ori-attB* in pACYC184; p15A*ori*; Ap^R^, Kn^R^ | This study |
| pTVC11 | | *pbad-rctB* in pGB2; pSC101*ori*; Sp^R^ | [4]; S10 Fig |
| pTVC13=p*rctB*LOW | | *pT7-rctB* in pET28a; pBR*ori*; Kn^R^ | [4] ; Figs 4,6,  S9 Fig |
| pTVC14=p*rctB* | | *pT7-rctB*-6XH in pET22b; pBR*ori*; Ap^R^ | [5]; Fig 5, S11 Fig |
| pTVC20=mini-Chr2 | | *ori2* (109–1,133) in R6K*oriγ*; Ap^R^ | [5] |
| pTVC25=mini-Chr2 |  | *ori2* (441-1133) in R6K*ori𝛾*; Ap^R^ | [5]; S11 Fig |
| pTVC31=mini‑Chr2 |  | *ori2* (775-1133) in R6K*ori𝛾*; Ap^R^ | [5] |
| pTVC210 |  | pTVC20 with *lacZ* from pMLB1109; *prctB-lacZ*; contains *rctA*; R6K*oriγ*; Ap^R^ | [6] ; S10 Fig |
| pTVC214 |  | pTVC210 with 5 bp insertion (5’-TACTG-3’) in nt 444 between 11-mer and 39-mer of *ig2*; R6K*oriγ*; Ap^R^ | [6] ; S10 Fig |

1. Malik A, Mueller-Schickert A, Bardwell JC. Cytosolic selection systems to study protein stability. J Bacteriol. 2014;196(24):4333-43. doi: 10.1128/JB.02215-14. PubMed PMID: 25266385; PubMed Central PMCID: PMC4248860.

2. Baek JH, Chattoraj DK. Chromosome I Controls Chromosome II Replication in *Vibrio cholerae.* PLoS Genet. 2014;10(2):e1004184. doi: 10.1371/journal.pgen.1004184. PubMed PMID: 24586205; PubMed Central PMCID: PMC3937223.

3. Jha JK, Li M, Ghirlando R, Miller Jenkins LM, Wlodawer A, Chattoraj D. The DnaK Chaperone Uses Different Mechanisms To Promote and Inhibit Replication of *Vibrio cholerae* Chromosome 2. MBio. 2017;8(2). doi: 10.1128/mBio.00427-17. PubMed PMID: 28420739; PubMed Central PMCID: PMCPMC5395669.

4. Pal D, Venkova-Canova T, Srivastava P, Chattoraj DK. Multipartite regulation of *rctB*, the replication initiator gene of *Vibrio cholerae* chromosome II. J Bacteriol. 2005;187(21):7167-75. PubMed PMID: 16237000.

5. Venkova-Canova T, Srivastava P, Chattoraj DK. Transcriptional inactivation of a regulatory site for replication of *Vibrio cholerae* chromosome II. Proc Natl Acad Sci U S A. 2006;103(32):12051-6. PubMed PMID: 16873545.

6. Venkova-Canova T, Chattoraj DK. Transition from a plasmid to a chromosomal mode of replication entails additional regulators. Proc Natl Acad Sci U S A. 2011;108(15):6199-204. Epub 2011/03/30. doi: 1013244108 [pii]10.1073/pnas.1013244108. PubMed PMID: 21444815.
