# Supplementary material for: Chromosome 1 licenses chromosome 2 replication in Vibrio cholerae by doubling the crtS gene dosage
Source: PLoS Genet. 2018 May 24;14(5):e1007426. doi: 10.1371/journal.pgen.1007426 (PMC5991422; doi:10.1371/journal.pgen.1007426)
Supplement: S3 Table — (DOCX) [file pgen.1007426.s017.docx]

**S3 Table Primers used in this study**

| **Name** | **Sequence** | **Used for amplifying** |
| --- | --- | --- |
| **RP017** | CAATCGCTTCTAAATAATGATTTAGTTACAACATACTTATTTTATATGTGCTGGGCCCAGCCGGCC | *terB-ori1-zeo-terB* from pGD101 for construction of CVC1157 |
| **RP018** | GGTTGGCTAGAAAATCGCTTTTTAGTTACAACATACTTATTTTATATCCTGTTTTTTCGATCAAGGAGGG |  |
| **RR03** | GTACACCCCATGTTCCTTTG | 1 kb upstream flanking region of *lacZ* from genomic DNA of CVC1121 for cloning into pRR06 |
| **RR04** | GAGCAGATCCTGTTCATGACGGTTCACC |  |
| **RR05** | GTCATGAACAGGATCTGCTCATGTTTGAC | *araC pbad*-*tus* from pBAD30-tus for cloning into pRR06 |
| **RR06** | GCCTTCGCGCGAAAAATAAACAAAAGAGTTTGTAG |  |
| **RR07** | GTTTATTTTTCGCGCGAAGGCGAAGCGGC | *lacI*^q^ *ptac*-(*tdTomato-*pMT*parB, gfp-*P1*parB t1t2*) from pRR01 for cloning into pRR06 |
| **RR29** | TAGTGTATGAGAGCGGATACATATTTGAATG |  |
| **RR09** | GTATCCGCTCTCATACACTAAATCAGTAAGTTGG | *cat* gene from pACYC184 for cloning into pRR06 |
| **RR10** | TTCGGGATGACGAATTTCTGCCATTCATC |  |
| **RR11** | GGTGTTCCACTCATCCCGAATTGGGGCA | 1 kb downstream flanking region of *lacZ* from genomic DNA of CVC1121 for cloning into pRR06 |
| **RR12** | GGTATTCATGCTCTGGCAACATTG |  |
| **RR40** | CACCGTCGCCGCCGTACGACTTGTACAGCTCGTCCATG | *tdTomato* from pALA2486 for cloning into pRR01 |
| **RR53** | TGTGGAATTGTGAGCGGCTCGAGGTTACGACGATGACGATAAGG |  |
| **RR41** | CGAGCTGTACAAGTCGTACGGCGGCGACGGTGATGTTAAA | *pMT:parB* from pALA2705 for cloning into pRR01 |
| **RR51** | GTGGAATTGTGAGCGGCTCGAGGTACGACG |  |
| **RR66** | CGGCCGCGGGAATTCGATGAACGTACGATCTGCACTT | 1 kb upstream of *ori1* from genomic DNA of CVC1157 for cloning into pRR08 |
| **RR72** | ATATAGGATGTTGTAACTAATATGATGAATACCTGAGGTCGTTC |  |
| **RR78** | ATATTAGTTACAACATCCTATATGAGACTATGGGTTAACAAAAGAGGCG | *terB-ori1-zeo-terB* from genomic DNA of CVC1157 for cloning into pRR08 |
| **RR79** | ATATTAGTTACAACATCCTATATCAATTAATCATCGGCATAGTATATCGGC |  |
| **RR80** | ATATAGGATGTTGTAACTAATATTCAACACGTGCTCGAGCTAGCGATATCG | 1 kb downstream of *ori1* from genomic DNA of CVC1157 for cloning into pRR08 |
| **RR71** | GATGAAGGTGGTGATTGCTGG |  |
| **RR101** | CATGGCGGCCGCGGGAATTCGATCGCGCGCAAGAGTTTACCACC | 805058 to 805991 from genomic DNA of CVC1121 for cloning into pRR16 |
| **RR102** | GTTTTACAACGTCGTGACTGGGAGAATTAATGATCTTCGCTTGCG |  |
| **RR103** | GTGAAATTGTTATCCGCTTAGCCTCGGTCGCTGCCGAGGTA | 806014 to 807194 from genomic DNA of CVC1121 for cloning into pRR16 |
| **RR104** | GCCGCGAATTCACTAGTGATACGCGCTTGTTGAAGCCACGCATG |  |
| **RR129** | AAGATCATTAATTCTCCTCAGAATCTGAGTGATC | *crtS* from genomic DNA of CVC1121 for cloning into pRR16 |
| **RR130** | CTGGGCCTCCATGTCTAACCGGATCTTCTGCTC |  |
| **RR131** | AGAAGATCCGGTTAGACATGGAGGCCCAGAATACCC | *nat1* gene from pAM101 for cloning into pRR16 |
| **RR132** | AGCGACCGAGGCTACAGTATAGCGACCAGCATTCAC |  |
| **RR140** | TCAGAATCTGAGTGAAAGTGAGAGGGAGTAGTGCCC | *attP* from pTF6 for cloning into pRR17 |
| **RR141** | GTGAAAAGATCACTAACGGCTTTGCCGCGGTACG |  |
| **RR142** | CTGGTCGCTATACTGTATTTCTAGACCGCGG | *attB* from pTF6 for cloning into pRR17 |
| **RR143** | GGATCTTCTGCTCCACTGAAAGGAGTACGCGC |  |
| **RR147** | CTACTCCCTCTCACTTTCACTCAGATTCTGAGGG | pBJH245 backbone and natural 1 kb flanks of *crtS* for cloning into pRR17 |
| **RR148** | CGTACTCCTTTCAGTGGAGCAGAAGATCC |  |
| **RR214** | CAGAAGATCCGGTTAGCGCGCAGAAAAAAAGGATCTC | *kanR* from pET28a for replacing *nat1* in pRR16 for cloning into pRR25 |
| **RR215** | CGGCAGCGACCGAGGCTACCAAATATGTATCCGCTCATG |  |
| **RR228** | TGCGCCAGAGTTGTTTCT | Marker frequency of *kanR* using qPCR |
| **RR229** | GATGGTCGGAAGAGGCATAAA |  |
